# The Hypoxic Proteome and Metabolome of Barley (*Hordeum vulgare* L.) with and without Phytoglobin Priming

**DOI:** 10.3390/ijms21041546

**Published:** 2020-02-24

**Authors:** Olga A. Andrzejczak, Jesper F. Havelund, Wei-Qing Wang, Sergey Kovalchuk, Christina E. Hagensen, Harald Hasler-Sheetal, Ole N. Jensen, Adelina Rogowska-Wrzesinska, Ian Max Møller, Kim H. Hebelstrup

**Affiliations:** 1 Department of Agroecology, Section of Crop Genetics and Biotechnology, Aarhus University, Forsøgsvej 1, DK-4200 Slagelse, Denmark; olga.andrzejczak@mbg.au.dk; 2 Department of Biochemistry & Molecular Biology and VILLUM Center for Bioanalytical Sciences, University of Southern Denmark, Campusvej 55, DK-5230 Odense M, Denmark; jhav@bmb.sdu.dk (J.F.H.); wwq0814@ibcas.ac.cn (W.-Q.W.); xerx222@gmail.com (S.K.); christinaeh@bmb.sdu.dk (C.E.H.); hasler@sdu.dk (H.H.-S.); jenseno@bmb.sdu.dk (O.N.J.); adelinar@bmb.sdu.dk (A.R.-W.); 3 Nordcee, Department of Biology, University of Southern Denmark, Campusvej 55, DK-5230 Odense M, Denmark; 4 Department of Molecular Biology and Genetics, Aarhus University, Forsøgsvej 1, DK-4200 Slagelse, Denmark; ian.max.moller@mbg.au.dk

**Keywords:** ethylene, anaerobiosis, hemoglobin, histones, N-end rule, stress priming

## Abstract

Overexpression of phytoglobins (formerly plant hemoglobins) increases the survival rate of plant tissues under hypoxia stress by the following two known mechanisms: (1) scavenging of nitric oxide (NO) in the phytoglobin/NO cycle and (2) mimicking ethylene priming to hypoxia when NO scavenging activates transcription factors that are regulated by levels of NO and O_2_ in the N-end rule pathway. To map the cellular and metabolic effects of hypoxia in barley (*Hordeum vulgare* L., cv. Golden Promise), with or without priming to hypoxia, we studied the proteome and metabolome of wild type (WT) and hemoglobin overexpressing (HO) plants in normoxia and after 24 h hypoxia (WT24, HO24). The WT plants were more susceptible to hypoxia than HO plants. The chlorophyll a + b content was lowered by 50% and biomass by 30% in WT24 compared to WT, while HO plants were unaffected. We observed an increase in ROS production during hypoxia treatment in WT seedlings that was not observed in HO seedlings. We identified and quantified 9694 proteins out of which 1107 changed significantly in abundance. Many proteins, such as ion transporters, Ca^2+^-signal transduction, and proteins related to protein degradation were downregulated in HO plants during hypoxia, but not in WT plants. Changes in the levels of histones indicates that chromatin restructuring plays a role in the priming of hypoxia. We also identified and quantified 1470 metabolites, of which the abundance of >500 changed significantly. In summary the data confirm known mechanisms of hypoxia priming by ethylene priming and N-end rule activation; however, the data also indicate the existence of other mechanisms for hypoxia priming in plants.

## 1. Introduction

As climate change progresses and urbanization rate increases, an increase in the incidence extreme weather conditions affecting people (2.3 thousand million people from 1995 to 2015, 95% of whom live in Asia) and arable land has been observed. Of all the weather-related disasters in recent years, up to 47% were related to flooding [1]. Flooding disproportionately affects Asia and Africa more than other continents, although increased flooding severity in Europe has also been observed. Recurrent flooding of agricultural land, particularly in Asia, has taken a heavy toll in terms of yield losses, food shortages, and rural malnutrition [2]. While it is not possible to estimate what percentage of flooding is due to climate change, current forecasts predict that the occurrence of flood-related disasters will continue to increase in the decades ahead [1].

Flooding limits oxygen availability for plants. Hypoxia occurs when oxygen levels decrease below the critical O_2_ pressure for respiration, and anoxia occurs when there is no oxygen [3]. Low oxygen accessibility also leads to a reduction in CO_2_ uptake, which inhibits photosynthesis and respiration. A limitation of underwater photosynthesis is the exponential decrease in light intensity as depth increases. Dissolved organic matter, small particles, and litter hinders light penetration in floodwater; consequently, net photosynthesis of submerged leaves is often significantly reduced [4]. In addition, hypoxia dramatically reduces the efficiency of cellular ATP production, which has diverse ramifications for cellular metabolism and developmental processes. The first response of plants to reduced ATP production is an increase in carbohydrate metabolism to generate ATP through glycolysis and to regenerate NAD+ through fermentation. In this process, enzymes, including alcohol dehydrogenase (ADH), pyruvate decarboxylase (PDC), lactate dehydrogenase (LDH), and Ala aminotransferase (AlaAT) play important roles. Knockout mutants of Arabidopsis show that plants lacking any of these enzymes are very sensitive to oxygen limitation [5].

Oxygen deprivation followed by reoxygenation results in the formation of reactive oxygen (ROS) and nitrogen species (RNS), and the amount of nitric oxide (NO) can increase 100-fold as compared with normoxia conditions [6]. NO plays an important role in many physiological processes including response to low temperature, heavy metals, salinity, drought, and disease resistance [7,8]. NO signaling is complex and involves various messenger molecules such as cGMP, cADP ribose, and Ca^2+^ [9], which can modulate (both directly and indirectly) signal transduction and modify the expression of specific genes. ROS includes molecules such as superoxide anion (O_2_^•−^), hydroxyl radical (HO^•^), and hydrogen peroxide (H_2_O_2_) [10,11]. Molecules of both types (ROS and RNS) are produced in the same cellular compartments and are involved in the plant’s response to the same environmental factors, therefore, their strong production and accumulation is described as nitro-oxidative stress, rather than two separate stresses [12,13,14].

The levels of ROS and NO are tightly controlled to prevent nitro-oxidative stress. ROS and NO can be removed by enzymatic antioxidant systems, such as the ascorbate-glutathione cycle [11], or by non-enzymatic reactions [15]. Most important for hypoxia is the reaction with phytoglobins (Pgb), previously called plant hemoglobins. The primary function of Pgb is to remove NO during the oxidation reaction of NO_3_^−^ through oxyhemoglobin. At the same time, reduction of NO_3_^−^ and NO_2_^−^ occurs, closing the Pgb/NO cycle. Reduced expression of Pgb1-1 in transgenic and mutant lines decreases plant growth and phenotypic abnormalities, in addition to increasing NO levels. Reduced NO content and overexpression of Pgb1-1, however, improved plant fitness during hypoxia [6,16]. NO acts as a plant signaling molecule in both biotic [8,17] and abiotic stress responses. During the abiotic stress response, NO increases resistance to drought, salinity, and oxidative stress [18,19]. Under normal physiological conditions, NO also plays an important role in plant development and metabolism [20], suggesting that control of NO levels in plants is required to activate signaling functions. NO is known to promote or antagonize H_2_O_2_-mediated signaling, or work alongside ROS signal transduction [21,22]. An important issue in the case of ROS and RNS cooperation is their ability to cause post-translational protein modification (PTM) [11,23]. PTMs can lead to the acquisition of new functions by the protein or loss of function; or PTMs can constitute as marker, directing the protein to the path of degradation or aggregation [24]. Furthermore, NO is involved as a signaling molecule in response to hypoxia. Overexpression of Pgb improves energy production in barley under low-oxygen stress, which leads to improved survival [25].

Pgb overexpression also leads to increased survival rate of plants in response to abiotic stresses such as salt [18], drought [26] and also in response to pathogen infection [27]. Barley plants with Pgb overexpression (HO) are characterized by stunted growth and decreased rate of germination [25,28]. However, they show much better germination rate under hypoxia conditions [25]. HO plants have significantly reduced levels of NO as compared with wild type (WT) plants under optimal conditions [26] and WT plants show two times higher NO accumulation than HO plants under hypoxia treatment [25]. The measurement of gene expression level of Pgb shows that HO plants have up to 70-fold higher expression than WT plant with simultaneous higher protein level [28].

Recently, it was reported by [29] that ethylene pretreatment (priming) improves plant survival to hypoxia by stabilizing the transcription factor ERFVII via induction of Pgb, and thus preventing a rise in the NO tissue level. NO and O_2_ are both involved in the N-end rule regulation of ERFVII. The removal of either NO or O_2_ increases the abundance of ERFVII. In this study, we used barley seedlings of the variety Golden Promise, without (WT) and with overexpression of phytoglobin (HO), to investigate the effect of increased phytoglobin levels on the proteome and metabolome of seedlings exposed to hypoxia. A comparison of waterlogging-sensitive and waterlogging tolerant genotypes of barley using 2-DE coupled with tandem mass spectrometry has previously identified 50 proteins in leaves with difference in abundance [30]. In this study, we used TMT labeled samples with 2D-LC-MS/MS to identify and quantify 9694 different proteins of which 1107 were significantly different in abundance between treatments or genotypes, and therefore were likely to be involved in low-oxygen response. The main objective was to identify differences that contribute to improved survival in the proteomic and metabolomic profiles of plants overexpressing Pgb; differences allowed us to identify the regulatory mechanisms involved.

## 2. Results

### 2.1. Physiological Differences between Genotypes in Response to Hypoxia Treatment

**Figure 1:**
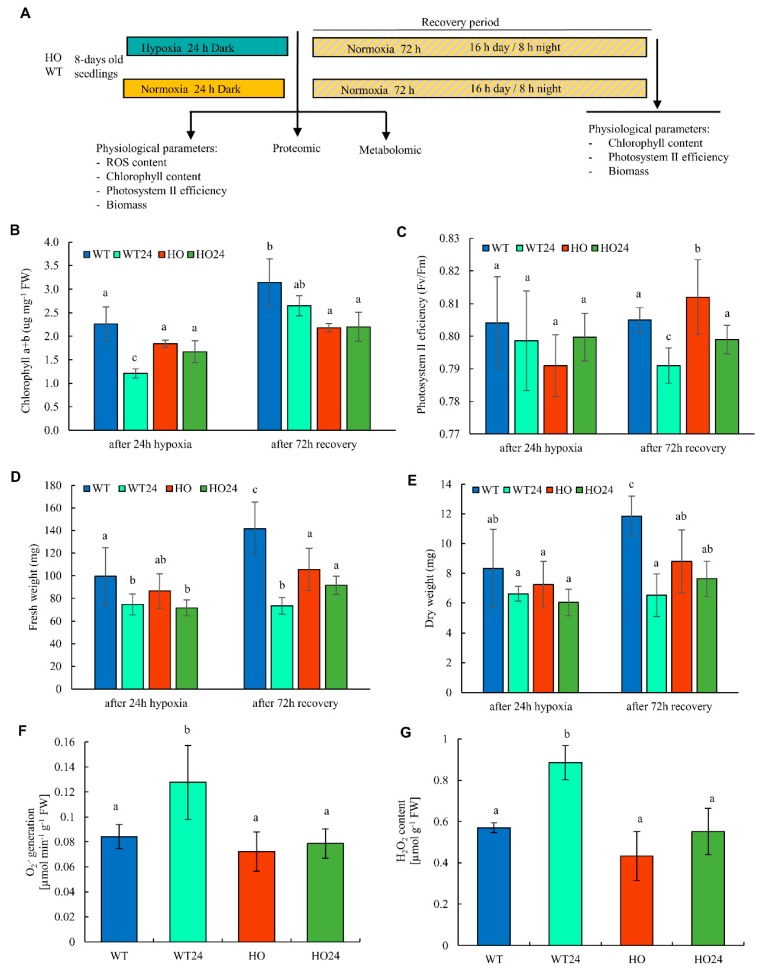
(**A**) Scheme of the experiments preformed. Eight-day-old seedlings of two genotypes were subjected to control (normoxia, wild type (WT), hemoglobin overexpressing (HO)) or hypoxic stress (WT24, HO24) for 24 h in darkness. Then, plants were kept for 72 h of recovery period in 16/8 h day/night conditions. The arrows indicate time of sampling for specific analysis. Changes in physiological parameters. (**B**) Chlorophyll content; (**C**) photosystem II efficiency; (**D**) fresh weight; (**E**) dry weight; (**F**) O_2_^•−^ generation; and (**G**) H_2_O_2_ concentration in barley leaves. Seedlings of wild type shown as WT and with overexpression of phytoglobin as HO, seedlings after hypoxia treatment shown as WT24 (wild type) and HO24 (overexpression of phytoglobin).

In our experiment, we used two genotypes that differ in the expression of phytoglobin 1 (Pgb) (Figure 1A). Lines with overexpression of the barley Pgb (formerly hemoglobin) gene *HvPGB1-1* (accession number: U94968), controlled by the maize ubiquitin2 promoter, are well characterized and have been used in other studies [25,27,28], which is why we decided to use only one representative line here. Seedlings were kept in darkness under hypoxia (WT24 and HO24) or normoxic conditions as controls (WT and HO). After 24 h, samples were taken for analysis, and the plants were kept for an additional 72 h recovery period, after which physiological parameters were measured again (Figure 1A).

In WT seedlings, hypoxia-induced changes were observed immediately after treatment. Chlorophyll a + b content was 50% lower in WT plants after hypoxia as compared with the control (Figure 1B), with no changes in photosystem II efficiency (Figure 1C). Fresh and dry weights of treated WT plants were 30% lower than the control (Figure 1D,E). During the recovery period, WT hypoxia plants were able to restore their chlorophyll levels, but not biomass (Figure 1B,D,E). In HO seedlings, physiological performance was not affected by the 24 h hypoxia treatment. Values for chlorophyll a + b, photosystem II efficiency, as well as fresh and dry weight, showed no difference between hypoxia-treated and control HO plants (Figure 1B–E). Thus, the physiological parameters of the two genotypes responded differently to hypoxia stress, indicating that the increased phytoglobin level (decreased NO level) in the HO plants gave a response similar to the priming effect observed after ethylene treatment [29].

It has been observed that there is a crosstalk between ROS and NO that allows them to work as signaling molecules under normoxia [31]. While both ROS and NO are produced during normal metabolism in plant cells, many abiotic factors can lead to increased production of different forms of ROS [31,32,33]. Standard metabolomic analyses do not cover small reactive molecules like ROS, and therefore they were measured separately. Under normoxia, both genotypes had the same tissue concentration of ROS, where H_2_O_2_ content was around 0.5 µmol·g^−1^·FW and O_2_^•−^ generation approximately 0.08 µmol·min^−1^·g^−1^·FW. In WT seedlings, the level of both molecules increased after hypoxia treatment (0.9 µmol·g^−1^·FW for H_2_O_2_ and 0.13 µmol·min^−1^·g^−1^·FW for O_2_^•−^) (Figure 1F,G). By contrast, ROS content was unaffected by hypoxia in HO seedlings. This observation is consistent with the idea that an increased NO level during hypoxia is a prerequisite for increased ROS levels.

### 2.2. Barley Leaf Proteome and Metabolome Affected by Hypoxia and Phytoglobin Overexpression

**Figure 2:**
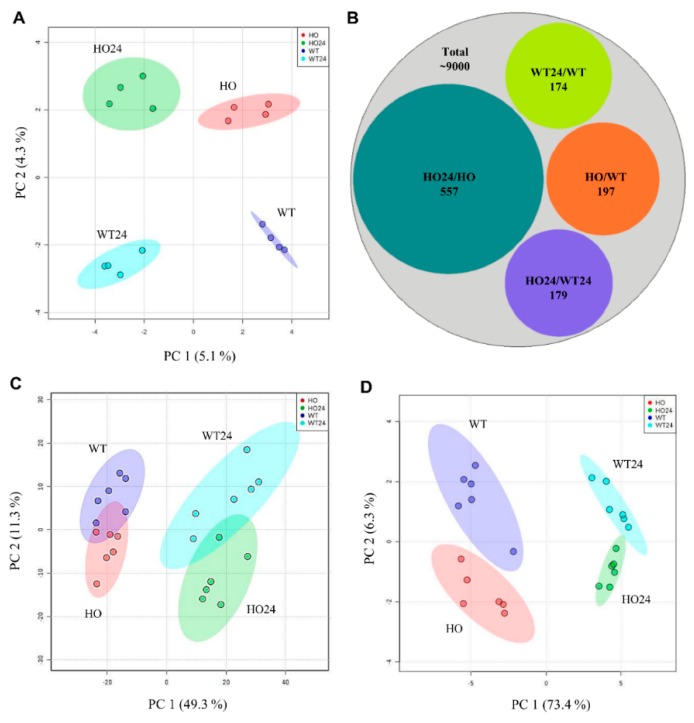
Changes in proteome and metabolome of barley seedling wild type (WT) and with overexpression of phytoglobin (HO) caused by hypoxia stress. (**A**) sPLS-DA of all the proteins identified in the experiment; (**B**) scheme showing the distribution of differentially accumulated proteins (DAP) between different experimental conditions in contrast to all identified proteins; PCA of the metabolite profiles of (**C**) all identified metabolites; and (**D**) selected metabolomics of amino acids, glycolysis pathway, and polyamines (list in Supplementary Table S4). PCA Vectors 1 and 2 were chosen for best visualization of differences between experimental treatments and include 9.4% (A), 60.6% (C), and 79.7% (D) of the information derived from proteomic and metabolic variance.

Proteomic analyses identified and quantified 9694 proteins (Table S1) in barley leaves, which were, then, analyzed by sparse partial least squares discriminant analysis (sPLS-DA) (Figure 2A) showing the variation between the factors with genetic variance explained by 4.3% of the proteins, and 5.1% of the treatment (hypoxia and normoxia) (Figure 2A). Afterward, the samples were analyzed as ratios between HO and WT (HO/WT) and between HO24 and WT24 (HO24/WT24) that showed the changes associated with the genetic component, or as HO24 and HO (HO24/HO) and WT24 and WT (WT24/WT) that display the hypoxia effect. Out of all the identified proteins, 1107 changed significantly in abundance between genotypes and treatments, so-called differentially abundant proteins (DAP) (Figure 2B and Table S2). The highest number of DAP (558) was observed in the HO plants after 24 h hypoxia treatment, i.e., in the HO24/HO comparison, whereas there were only 174 DAP in the WT24/WT (Figure 2B).

Furthermore, we identified and quantified 1470 different metabolites in barley leaf extracts (Table S3). The principal component analysis (PCA), based on 1470 metabolites, showed that 49.3% of the variance was associated with the hypoxia treatment (PC1 in Figure 2C), while 11.3% was associated with the genotype (PC2 in Figure 2C). The metabolites that had the largest impact on the observed variance were connected with glycolysis, Krebs cycle, and amino acids and were selected for further analysis (Table S4 and Figure S1). PCA in Figure 2D illustrates the variance between experimental treatments of selected metabolites. All three PCAs show that hypoxia had a larger overall effect on the metabolome and proteome than overexpression of phytoglobin (Figure 2A,C,D); however, for certain proteins (see next section) and metabolites, e.g., phosphoglyceric acid (Figure S1), the genotype effect was dominant.

### 2.3. Specific Proteins Changing in Abundance in Seedlings with Overexpression of Pgb

**Figure 3:**
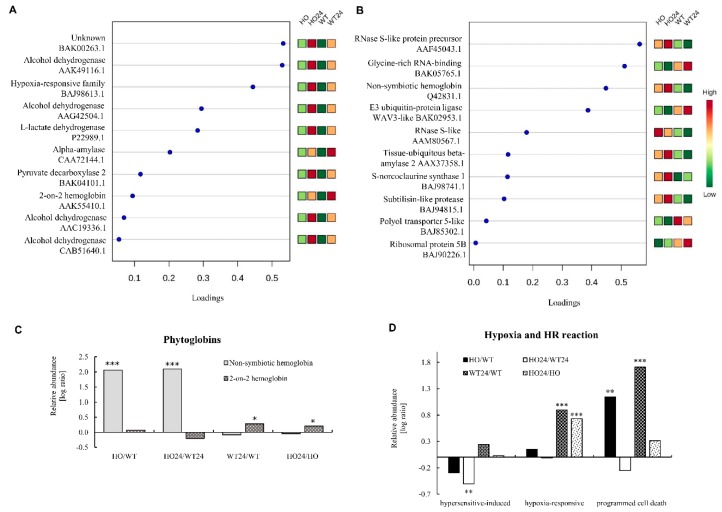
Proteins having the major impact on the differences caused by (**A**) hypoxia and (**B**) genotype. Colors indicate high (red) or low (green) abundance of protein; (**C**) specific proteins confirming overexpression of phytoglobin; (**D**) hypoxia stress and different reaction to stress of two genotypes of plants. Asterisk indicate level of p (* *p* < 0.05, ** *p* < 0.01, and *** *p* < 0.001).

To confirm that the HO plants produced more Pgb, we looked for the protein among the DAP. Both HO and HO24 seedlings produced the same higher amount of overexpressed non-symbiotic hemoglobin (Q42831.1) (now called phytoglobin) (Figure 3C). Surprisingly, we also found a second type of Pgb (two-on-two hemoglobin, AAK55410.1) that increased after hypoxia treatment but was present at the same level in both genotypes (Figure 2A). We know that NO production by barley plants can increase 100-fold during hypoxia [6], therefore, the plants could need more than one hemoglobin protein to regulate the cellular NO concentration.

We also checked the loadings from sPLS-DA of proteins to see which 10 proteins had the largest impact on variance between experimental treatments. Loadings for Component 1 that showed the hypoxia effect were, as expected, mostly alcohol dehydrogenases, L-lactate dehydrogenase, and pyruvate decarboxylase (Figure 3A), which are related to changes in energy status during hypoxia. Among them, we also found hypoxia-responsive family protein (BAJ98613.1) and the two-on-two hemoglobin (AAK55410.1) mentioned above. The former increased strongly in abundance (Figure 3B), further confirming that the hypoxia treatment worked. and the second indicated that Pgb plays a very important role during low oxygen stress. Interestingly, the largest difference between hypoxia and normoxia was due to unknown protein (BAK00263.1), that is, 97 amino acids protein without any specific domains. Using BLAST, we found more than 90 similar proteins in different species, all of them uncharacterized. The most similar from *Arabidopsis thaliana* was hypothetical protein AT5G65207 (score 31.6, *E* value 0.049, and 72% identity). This protein is 73 amino acids long and does not have any specific domains, but it is annotated as “hypoxia response” because its expression changed during hypoxia and reoxygenation [34]. The unknown barley protein could be important for oxygen sensing and response to hypoxia.

Non-symbiotic hemoglobin (Q42831.1) that was overexpressed in HO plants and also proteins connected with protein and RNA degradation, secondary metabolism, and ribosomal protein were among the top ten loadings responsible for the variance between genotypes (Figure 3B). S-like RNase that was upregulated in HO seedlings, but not in WT seedlings, is known to be expressed under certain environmental conditions and participate in nutrient recycling processes during senescence and nutrient starvation [35].

There were also significant genotype-associated changes in the programmed cell death protein (BAK04024.1) that is a marker for PCD, which increased in abundance in WT24/WT, but not in HO24/HO (Figure 3D). Furthermore, a marker for the hypersensitive reaction (HR) (hypersensitive-induced protein, AAN17464.1) decreased in abundance in HO24/WT24 (Figure 3D), indicating that NO contributes to signaling in the PCD process.

### 2.4. Functional Analysis of Differentially Abundant Proteins

**Figure 4:**
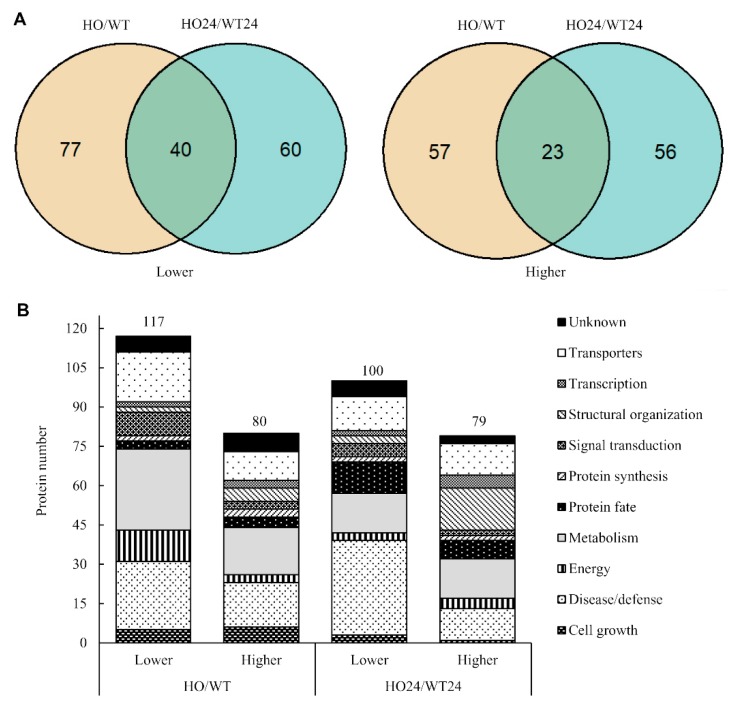
Genotype differences in the leaf proteome. (**A**) Comparison of the number of differentially abundant proteins (DAP) in HO/WT and HO24/WT24 seedlings whose quantity was decreased (lower) or increased (higher); (**B**) the functional distribution of the DAP proteins whose abundance was decreased (lower) or increased (higher) in HO/WT and HO24/WT24.

**Figure 5:**
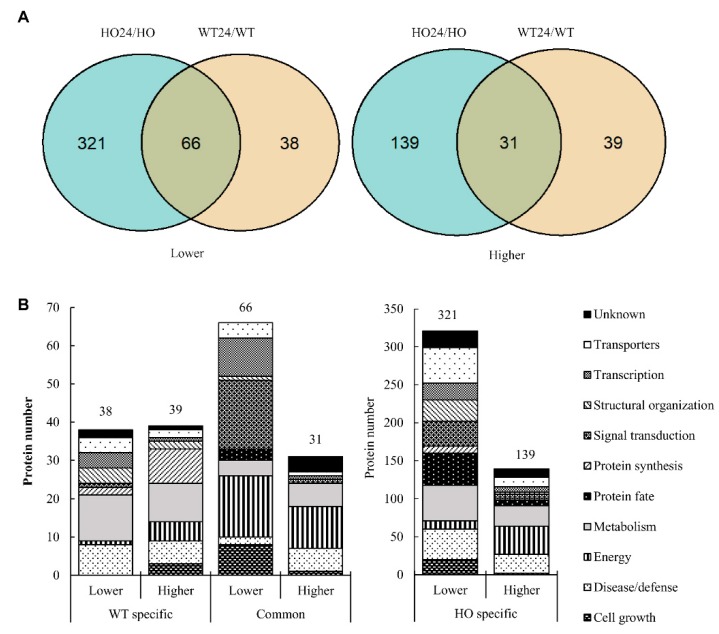
Hypoxia differences in the leaf proteome. (**A**) Comparison of the number of DAP that were accumulated in higher and lower abundance in HO24/HO and WT24/and the common proteins between them; (**B**) comparison of the functional categories of DAP that were accumulated in higher and lower abundance specifically in HO and WT plants after hypoxia and the common between them.

We observed that in HO seedlings as compared with WT, more DAP decreased in abundance (117 before and 100 after hypoxia) than increased (80 and 79, respectively) (Figure 4A). For a functional overview, the DAP were separated into 11 categories, based on their functions (Figure 4B), using the UniProt database according to the category list in [36]. The number of DAP in each of the functional groups varied depending on the genotype (Figure 4B) and response to hypoxia treatment (Figure 5B). The largest number of DAP had a function related to metabolism (220), while the lowest was related to protein synthesis (20) (Figure 4B and Figure 5B). More metabolism-related DAP changed their abundance in HO/WT before hypoxia (49) than after hypoxia (30). Similarly, for proteins related to transporters, the number of DAP changed from 30 in HO/WT to 24 HO24/WT24. However, the number of DAP increased in HO24/WT24 as compared with HO/WT for proteins related to the structural organization (from seven in HO/WT to 19 in HO24/WT24), disease/defense (from 43 to 48), and protein fate (from seven to 19) (Figure 4B). Thus, the modification of phytoglobin content affected protein abundance both before and after hypoxia.

Considering the proteomic response to hypoxia, a greater number of DAP was found for HO24/HO than for WT24/WT (558 and 174, respectively) (Figure 5A). Out of these, the vast majority (460) was found only in HO/HO24, 77 only in HO24/WT24, whereas 97 were found in both. Energy-related DAP was predominantly found in the common category (27 proteins) and in the HO-specific category, where they mostly increased (37 proteins increased and 11 decreased) (Figure 5B). In the signal transduction category, WT-specific DAP were hardly found (one protein), while many common (18 proteins) and HO-specific DAP (33 proteins) were observed to decrease. Many HO-specific transporters (47 proteins) decreased in response to hypoxia (Figure 5B).

Both ROS and NO play important roles in signaling processes leading to PCD. Overexpression of phytoglobins can disturb the PCD and HR responses of plants. We observed an increased intensity of PCD in the form of the changed amount of hypersensitive-induced response protein (AAN17464.1) and programmed cell death (BAK05821.1) in WT seedlings exposed to low oxygen content (Figure 2D). In PCD, both NO and ROS concentration must be at a specific level to fulfill their function as signal molecules. NO can regulate its concentration by the feedback of nitrogen flux through nitrite assimilation pathways and, furthermore, controls its bioavailability by modulating its consumption by the posttranslational modification of scavenging enzymes [33]. In Arabidopsis, it was shown that NO regulates its radicals such as ONOO^−^, as well as ROS through tyrosine nitration of peroxiredoxin II E (PRDX IIE). This leads to obstruction of PRDX IIE detoxifying activities [37]. The reduced amount of NO in plants with overexpression of phytoglobin (in barley as measured by [25] and [26]) probably causes disturbances in signal transduction by both NO and ROS, and thus affecting PCD.

In the following sections, we present and briefly discuss DAP belonging to several functional groups of proteins, mostly enzymes, and their behavior in response to genotype and hypoxia treatment. Metabolomic data is integrated into the discussion where appropriate.

### 2.5. Energy-Related Proteins and Metabolites

Plants exposed to O_2_-deficient conditions must overcome the restriction of mitochondrial oxidative phosphorylation. To do this, plants use the fermentative pathways, involving enzymes such as alcohol dehydrogenase and pyruvate decarboxylase, which are far less energy-efficient and cause accumulation of metabolites such as ethanol and lactate [38].

**Figure 6:**
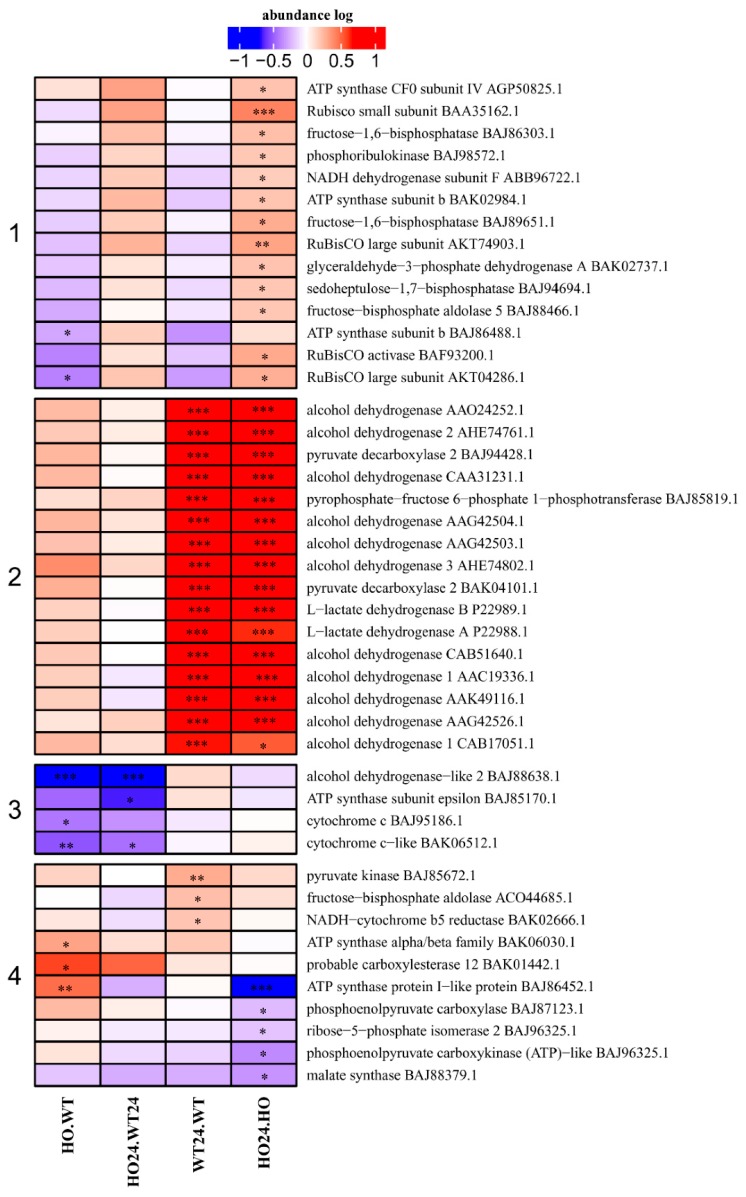
Heatmap displaying the abundance of DAP related to energy function. Seedlings of wild type shown as WT and with overexpression of phytoglobin as HO, seedlings after hypoxia treatment shown as WT24 (wild type) and HO24 (overexpression of phytoglobin). The color scale illustrates the relative abundance level of each protein across the 3 biological samples; red and blue indicate higher and lower expression in comparisons. Heatmap is showing 4 largest clusters created with k-means. Asterisk indicate level of *p* (* *p* < 0.05, ** *p* < 0.01, and *** *p* < 0.001).

DAP with function related to energy, were divided into four subcategories (Figure 6). In Cluster 1 contained proteins that had increased abundance in HO24/HO. These proteins were connected to the Calvin cycle, electron transport chain, and glycolysis. The DAP in Cluster 2 were all involved in fermentative metabolism and were all markedly increased in abundance in both WT24/WT and HO24/HO. This is further evidence that the hypoxia treatment worked and that the operation of the fermentation pathways was induced in both genotypes. Cluster 3 contained proteins with decreased abundance in the HO/WT or the HO24/WT24 comparisons. There were proteins connected to the electron transport chain (cytochrome c and ATP synthase subunit epsilon) and one alcohol dehydrogenase-like (BAJ88638.1). In Cluster 4 were placed proteins that had decreased abundance in HO24/HO, or higher in WT24/WT, or higher in HO/WT, but all related to the glyoxylate cycle, pentose phosphate pathway, glycolysis, or electron transport chain (Figure 6).

Among glycolysis-related protein, in plants with Pgb overexpression, a significantly higher content of glyceraldehyde-3-phosphate dehydrogenase (GAPDH, BAK02737.1) was observed. GAPDH plays an important role in animals, bacteria, and plants. GAPDH gene expression is often assumed to be unaffected by external factors [39], but the abundance of the protein changed in barley during hypoxia (Figure 6). During the cellular response to hypoxic stress, GAPDH acts as a stress protein and activates the expression of hypoxia-inducible factor-1 (HIF-1) [40], indicating that GADPH can enhance the protective effect on cells by promoting hypoxic metabolism. Interestingly, one of alcohol dehydrogenase-like 2 (ADH2 like BAJ88638.1) (Figure 6, cluster 3) was significantly lower in HO plants, both before and after the hypoxia treatment (HO/WT and HO24/WT24). This particular enzyme has not been tested in experimental conditions in barley so far, but it could have a slightly different substrate specificity than the other ADH we found. It could be, for example, S-nitrosoglutathione reductase (GSNOR), which according to the enzyme classification is part of the family of Zn-dependent class III alcohol dehydrogenases (EC 1.1.1.1), which are distinct from members of class I ADH family by their high affinity towards long-chain alcohols such as cinnamyl alcohol, farnesol, and geraniol. The main function of GSNOR is the reduction of S-nitrosoglutathione (GSNO) [41]. Increased expression of Pgb results in a decreased availability of free NO, and thus a reduced amount of GSNO. Additional studies are needed to establish this point. A study by [25] reported that GSNOR activity increased in barley with overexpression of Pgb during anoxia.

In the case of metabolites associated with energy production, we only observed two significantly changed metabolites involved in glycolysis-fructose-6-phosphate and 3-phosphoglyceric acid, the first increased under the hypoxia treatment in both HO and WT plants, and the second was characterized only by the difference between genotypes (increased in HO24 but not in WT24) (Figure S1B). Additional differences were found in the amount of pyruvic acid and lactic acid, where both were increased by hypoxia, and both were lower in WT24 than in HO24 (Figure S1B).

### 2.6. Transport-Related Proteins

**Figure 7:**
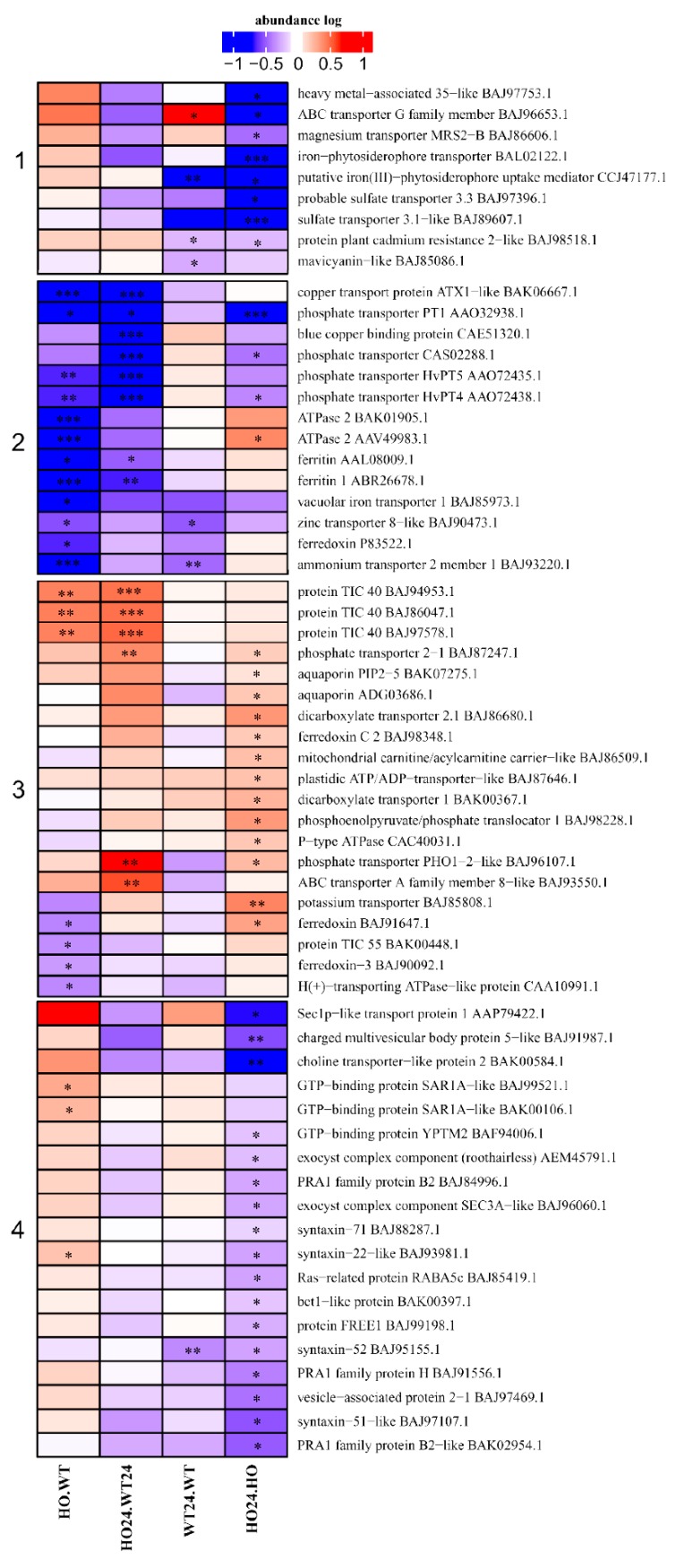
Heatmap displaying DAP related to transport. Seedling of wild type shown as WT and with overexpression of phytoglobin as HO, seedlings after hypoxia treatment shown as WT24 (wild type) and HO24 (overexpression of phytoglobin). The color scale illustrates the relative abundance level of each protein across the 3 biological samples; red and blue indicate higher and lower expression in comparisons. Heatmap is showing 4 largest clusters created with k-means. Asterisk indicate level of *p* (* *p* < 0.05, ** *p* < 0.01, and *** *p* < 0.001).

We observed changes in the amount of transport proteins that were characteristic for HO plants, but their abundance was still lower under the influence of hypoxia (Figure 7, Group 2). Group 1 proteins (Figure 7) had a lower abundance in HO24/HO and were associated with ion transport (Zn^2+^, Cu^2+^, and Fe^2+^). The reduced amount of the transporters could be related to a lower amount of NO and ROS in HO plants, and thus to disturbed signaling. Studies have shown that stress caused by an increased amount of ROS can affect the content of Zn^2+^, Cu^2+^, and Fe^2+^, at the same time these ions operate in a kind of ”stress feedback loop”, where accumulated Zn^2+^, Cu^2+^, and Fe^2+^ additionally stimulate the production of ROS [42,43]. The Group 3 proteins (Figure 7) were mostly present at higher abundance in HO24/HO seedlings. They were related to the transport of cations, phosphate, and water (aquaporin PIP-2, BAK07275.1, ADG03686.1). Interestingly, HO plants seem to be generally better adapted to stress associated with the availability of water [26]. Flooding or hypoxia often reduces water availability to plants. Both PIP1 and PIP2 aquaporin possess a histidine residue (HIS197), which is protonated at low pH causing the channel to close. This hinders water influx into the cell during oxygen deprivation [44]. Simultaneously, the amount of transporters decreased only in the hypoxia-treated seedlings, WT24/HO24, whereas, in the HO/WT seedlings, their abundance was unchanged (Figure 7, Groups 1 and 4). In this group were sulfate, ion, and vesicle trafficking along with transporters responsible for maintaining the proton gradient. Low oxygen availability can lead to acidification of the cytoplasm due to proton release through ATP hydrolysis, as well as from the low ATP concentration reducing the activity of the plasma membrane proton pumps [44]. During prolonged hypoxia, the maintenance of the membrane potential is compromised, and depolarization of the plasma membrane leads to K^+^ loss [45].

### 2.7. Signal Transduction-Related Proteins

**Figure 8:**
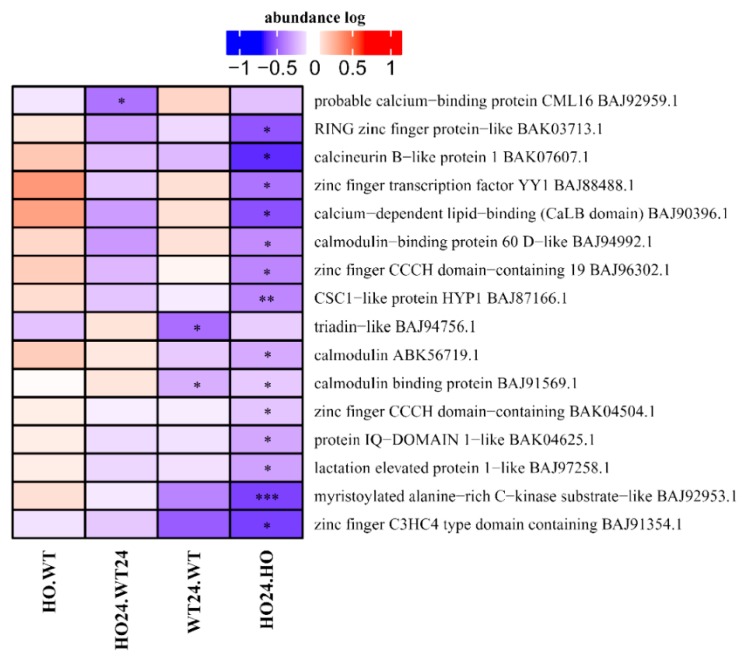
Heatmap displaying DAP related to calcium ion signal transduction. Seedling of wild type shown as WT and with overexpression of phytoglobin as HO, seedlings after hypoxia treatment shown as WT24 (wild type) and HO24 (overexpression of phytoglobin). The color scale illustrates the relative abundance level of each protein across the 3 biological samples; red and blue indicate higher and lower expression in comparisons. Asterisk indicate level of *p* (* *p* < 0.05, ** *p* < 0.01, and *** *p* < 0.001).

Ca^2+^ is an important second messenger that activates downstream signaling components associated with the response to low oxygen stress [46]. Among DAP related to signal transduction, Ca^2+^ signaling-related proteins constitute the largest group (Figure 8), however, it was not subdivided into clusters because the group only contained 16 DAP. Despite this, the group is quite homogeneous. Most DAP were characterized by a decrease in abundance in HO24/HO, and only the probable calcium-binding protein CML16 (BAJ9295.1) was downregulated in HO24/WT24, triadin-like (BAJ94756.1) was lower in abundance in WT24/WT, and carmodulin binding protein (BAJ91569.1) had lower abundance in both HO24/HO and WT24/WT (Figure 8).

Hypoxia depolarizes the mitochondrial membrane due to the restricted electron transport chain (ETC) to induce Ca^2+^ release from the mitochondria to the cytosol [47]. Under hypoxic conditions, depolarization of the plasma membrane leads to Ca^2+^ influx into the cytosol. Then, a higher concentration of Ca^2+^ in the cytosol is detected by Ca^2+^-sensing proteins such as calcineurin B-like (CBL)/Ca^2+^-independent protein kinases (CIPKs), calmodulin (CaM), calmodulin-like proteins (CMLs), and Ca^2+^-dependent protein kinases (CDPKs) [47]. We observed a decreased amount of proteins associated with Ca^2+^ signal in plants with overexpression of Pgb as compared with in the WT plants (HO24/HO) (Figure 8). Hypoxia-induced Ca^2+^ changes are highly species and tissue (organ) specific [46]. By comparing hypoxia-induced changes in Ca^2+^ in rice and wheat roots, it was concluded that both external and internal Ca^2+^ stores were important for stress-induced Ca^2+^ elevation in rice, whereas the hypoxia-intolerant wheat did not require an external source for the rise in Ca^2+^ levels. Changes in the cellular Ca^2+^ concentration appear to be an important component of the low oxygen-sensing mechanism.

### 2.8. DAP with Function Connected to Protein Fate

**Figure 9:**
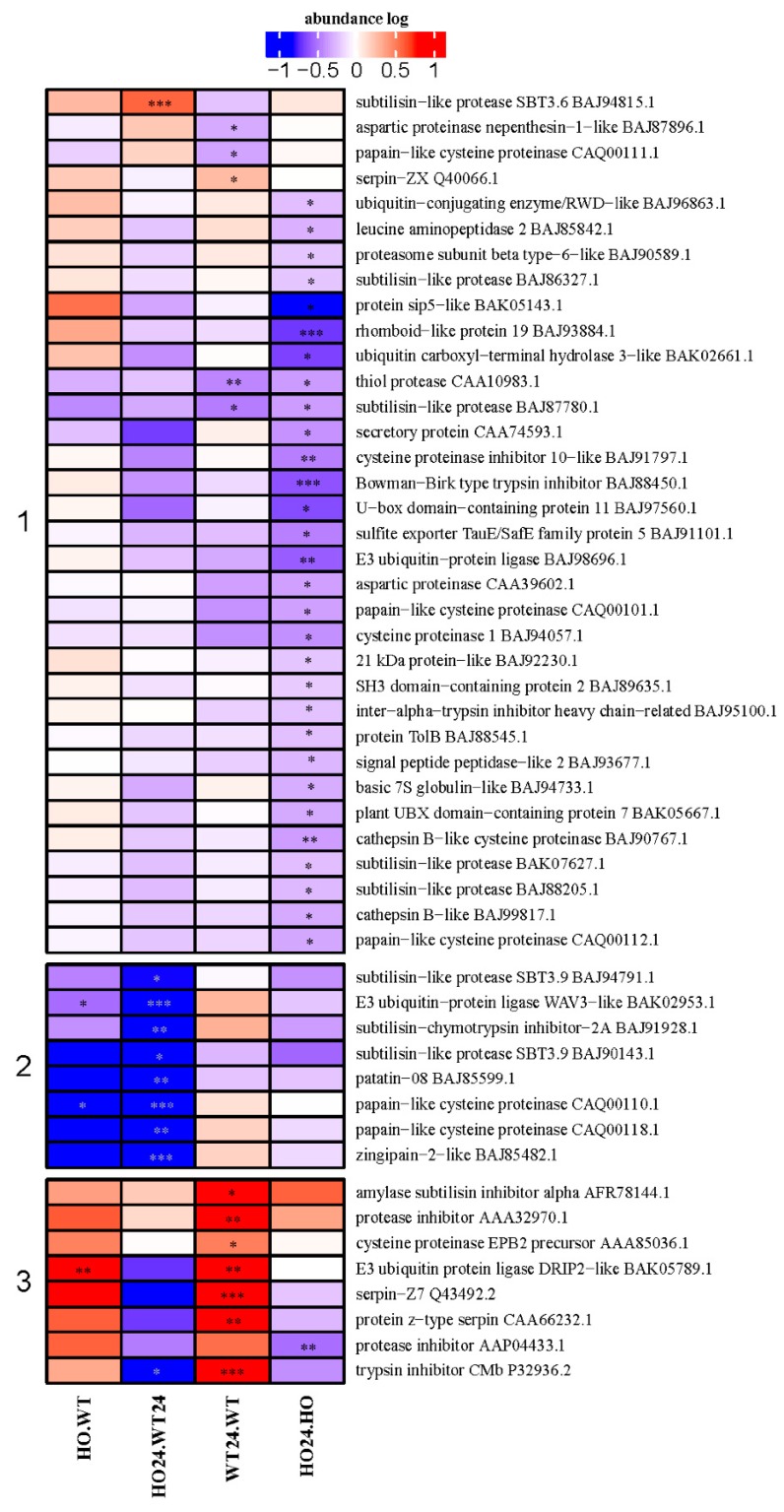
Heatmap displaying DAP with function related to protein fate. Seedling of wild type shown as WT and with overexpression of phytoglobin as HO, seedlings after hypoxia treatment shown as WT24 (wild type) and HO24 (overexpression of phytoglobin). The color scale illustrates the relative abundance level of each protein across the 3 biological samples; red and blue indicate higher and lower expression in comparisons. Heatmap is showing 3 largest clusters created with k-means. Asterisk indicate level of *p* (* *p* < 0.05, ** *p* < 0.01, and *** *p* < 0.001).

In the group of proteins with function related to protein fate, it was possible to distinguish three clusters. Cluster 1 was the largest with proteins that decreased in abundance in HO24/HO. In Cluster 2 were DAP with a lower abundance in HO24/WT24. In the last cluster were DAP with higher abundance in WT24/WT (Figure 9). The proteins with function related to protein fate were associated with proteolysis, tagging for proteolysis or inhibitors of proteases.

The lower abundance of proteases in HO seedlings under hypoxia (Figure 9, Groups 1 and 2) is probably related to the change in signal transduction by NO. Proteases are implicated in all cellular processes. Some of them are involved in maturation of other proteins and some perform degradation to ensure the maintenance of protein complexes and contributing to the recycling of nitrogen [48]. Among them was the member of the Bowman–Birk (BBirc) protein family, which are serine protease inhibitors, widely distributed in leguminous and cereal plants. In rice, these proteases are rapidly induced by jasmonic acid, ethylene, and wounding [49]. Rice seedlings grown under hypoxia conditions had an increased content of BBirc [49], and the correlation of BBirc abundance with reoxidation after hypoxia stress indicated its role in the regulation of ROS-induced damage. It is also likely that the BBirc proteins are involved in the scavenging mechanism of free radicals due to their large number of disulfide bridges [50]. Protein degradation in plants is a complex process involving a multitude of proteolytic pathways that can be carried out in different cell compartments, but it is also associated with oxidative stress promoted by ROS. One of the effects of ROS overproduction could be carbonylation of proteins (formation of a carbonyl group on the side chain of amino acids in protein). Then, carbonylated proteins are moved to proteolysis or aggregation [32,51]. Higher levels of ROS in WT barley seedling could cause a higher level of some proteases observed after hypoxia (Figure 9, Group 3). However, it can also be associated with programmed cell death (PCD) in which proteases play roles of great importance.

### 2.9. Proteins Connected to Hormone and Polyamine Metabolism or Transport

**Figure 10:**
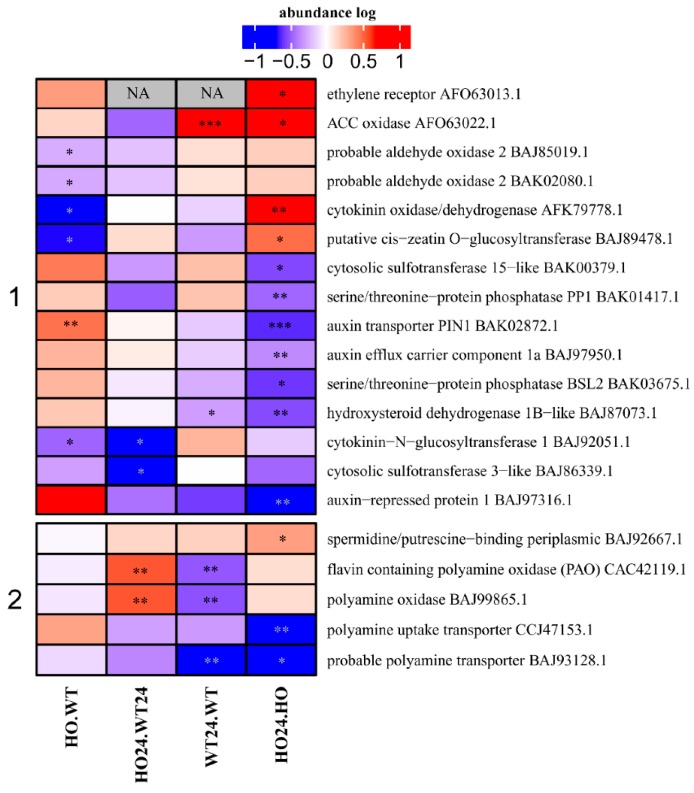
Heatmap displaying DAP connected with (**1**) hormone metabolism/transport and (**2**) polyamines. Seedling of wild type shown as WT and with overexpression of phytoglobin as HO, seedlings after hypoxia treatment shown as WT24 (wild type) and HO24 (overexpression of phytoglobin). The color scale illustrates the relative abundance level of each protein across the 3 biological samples; red and blue indicate higher and lower expression in comparisons. Asterisk indicate level of *p* (* *p* < 0.05, ** *p* < 0.01, and *** *p* < 0.001).

Changes in the abundance of proteins involved in hormonal signaling were also observed. This group of proteins was divided into two clusters according to their function (Figure 10). Proteins in Cluster 1 are related to hormones and proteins in Cluster 2 are related to polyamines. Among hormones were proteins connected to auxin (IAA), cytokinins (CK), gibberellins (GA), and ethylene (Eth). Different DAP related to hormones show different patterns of abundance. Auxin transporters (PIN1, BAK02872.1) decreased in HO24/HO. By comparison, a higher level of PIN1 was observed in HO/WT (Figure 10). At the same time, both HO24/HO and WT24/WT plants showed higher levels of ACC oxidase, involved in ethylene biosynthesis. It could be related to the induction of gene expression under hypoxia that requires transcription factors belonging to group VII ethylene response factors (ERF-VII). ERF-VII depend on ethylene, which stimulates Pgb expression and, eventually, leads to lower NO production and stabilization of transcription factors [52]. However, no gene products activated by ERF-VII (RAP-type, HRE-type) were detected among the proteins identified and quantified.

In Cluster 2, transporters of polyamines decreased in abundance in HO24/HO and in WT24/WT. However, the polyamine oxidases had a higher abundance in HO24/WT24 and lower in WT24/WT at the same time (Figure 10). Polyamines (PAs) are polycations involved in many processes of plant growth and development and are well known for their anti-senescence and anti-stress effects [53]. However, they can also affect NO production [54]. We observed that the amount of putrescine depended on the genotype, in HO, it was much lower than in WT (Figure S1).

### 2.10. DAP Related to Chromatin Organization

**Figure 11:**
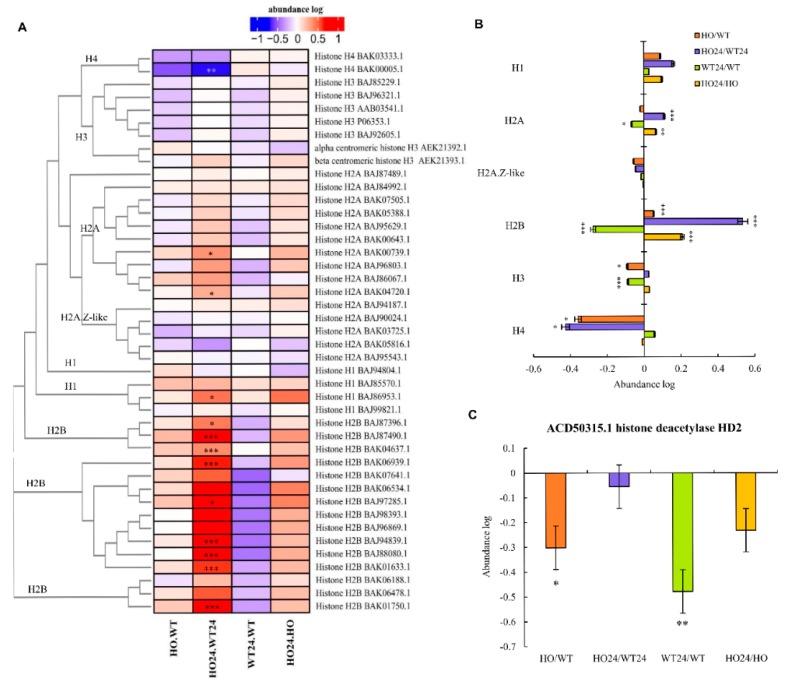
Changes in abundance of histones. (**A**) Heatmap displaying all histones found in proteomic analysis with phylogenetic tree of sequences; (**B**) abundance of pulled histones of the same type, showing differences between experimental treatments; (**C**) changes in abundance of histone deacetylase. Seedling of wild type shown as WT and with overexpression of phytoglobin as HO, seedlings after hypoxia treatment shown as WT24 (wild type) and HO24 (overexpression of phytoglobin). The color scale illustrates the relative abundance level of each protein across the 3 biological samples; red and blue indicate higher and lower expression in comparisons. Heatmap is showing asterisk that indicate level of *p* (* *p* < 0.05, ** *p* < 0.01, and *** *p* < 0.001) in ratio, and graph (B) asterisk indicate significance from 0 by one sample T-test.

Gene expression during hypoxic stress is regulated partly by chromatin restructuring involving histone modification [34]. We identified 43 proteins, either annotated as histones or with high similarity to other histones (Figure 11A). Their phylogeny was analyzed by Clustal Omega [55]. The resulting phylogenetic tree shows that they classify well into the five histone types, H1, H2A, H2B, H3, or H4. The level of each group was pooled, and the log ratios tested for significant difference from 0 (Figure 11B). The level of H2B histones collective showed a tendency to be upregulated in HO and downregulated by hypoxia in the wild type plants. However, in the HO line, their level was rather upregulated by the hypoxic treatment, so that a very significantly higher level of H2B histones was found in hypoxic HO plants as compared with hypoxic WT plants. A similar, but less pronounced pattern was found for H3 and H2A histones, except those of the H2A.Z-like type. In addition, H1 histones and H4 histones showed a different pattern in response to hypoxia and genotype.

Changes in DNA binding to different histone types by acetylation and methylation of lysine residues in the histones is a known response to stress and an important regulatory mechanism in gene expression [56]. Peptides that are modified by acetylation or methylation were not detected in our analyses, and changes solely in the ratios of modified/unmodified histones could, therefore, appear as changes in the total levels of the proteins. To test if the changes in histone levels that we observed could be due to acetylation or methylation, we co-mapped identified peptides and amino acid sites known to be targets for acetylation of methylation in each histone (Supplementary Figure S6). This showed that most of the identified peptides are not known to be targets for acetylation or methylation. Our results, consequently, suggest that hypoxia results not only in modification but also in changes in abundance of different histone isoforms and that phytoglobins interfere with this response. In Arabidopsis, NO inactivates histone deacetylases by the formation of –SNO groups, and thus indirectly regulates histone acetylation [57]. Our data suggest that in addition to regulation of histones deacetylates through NO, expression of phytoglobin can also cause changes in the abundance of histone acetylase (Figure 11C).

### 2.11. Changes in the Content of Amino Acids

**Figure 12:**
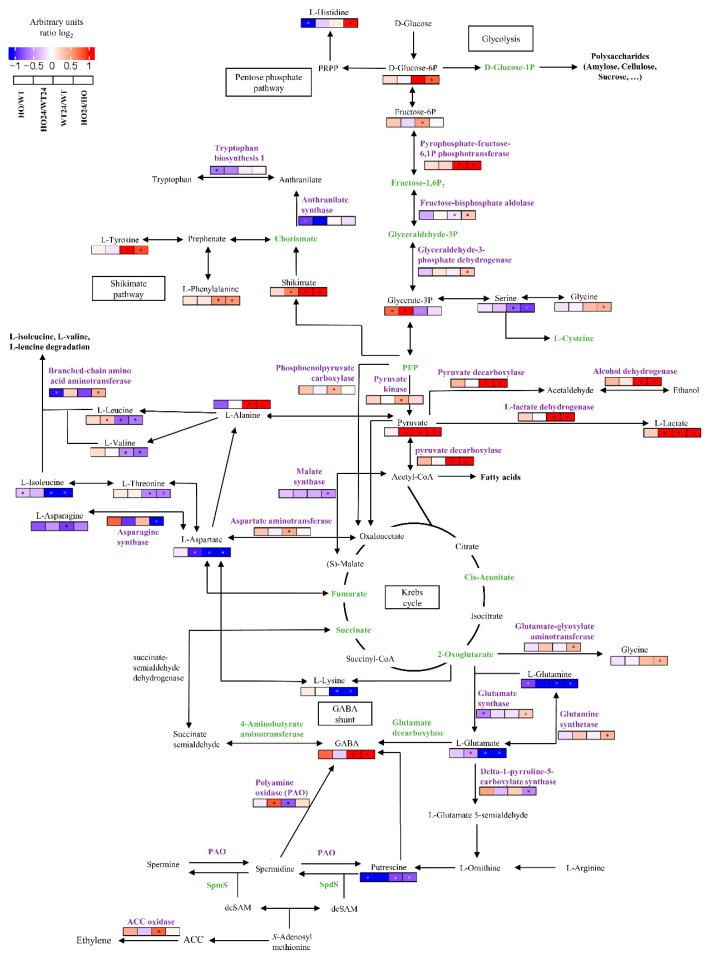
Diagram showing condense metabolic pathways of glycolysis, Krebs cycle, amino acids, polyamines, and gamma-aminobutyric acid (GABA) shunt with marked metabolites and enzymes (purple) that were significantly different for different treatments. The color scale illustrates the relative log_2_ units of each metabolite or protein across the 4 biological samples (HO, overexpression of phytoglobin; WT, wild type; HO24 and WT24, hypoxia treatments); red and blue indicate higher and lower level. Metabolites and proteins in green were identified and quantified but did not show significant change between treatments. The pathways are based on KEGG, data in Supplementary Table S5.

Among the selected metabolites, amino acids constituted a particularly important group. The content of 12 amino acids changed significantly (Figure 12 and Figure S1A). In both genotypes, half of the amino acid (Asp, Glu, Gln, Ile, Lys, Ser) showed a reduced amount, and half (Ala, Gly, Tyr, Phe, Pro, His) showed an increased amount after hypoxia treatment. Only Pro and His showed a significant difference between the two genotypes. Similar changes in amino acid content were observed previously in plants under low oxygen conditions in soybean [58], but also in response to other stresses [59].

### 2.12. Disease/Defense-Related Proteins

Proteins with functions related to diseases/defense were divided into three subgroups, defense-related (Figure S2), stress response (Figure S3), and detoxification (Figure S4).

In the defense-related group, three clusters were separated (Figure S2). Cluster 1 contains proteins that were less abundant in both HO/WT and HO24/WT24 or only HO24/WT24. Cluster 2 contained proteins that increased in abundance in HO24/HO, WT24/WT or HO/WT. Cluster 3 was the largest and contained proteins that decreased in abundance in HO24/HO (Figure S2). Interestingly, there was a decreased amount of JAR2 (jasmonic acid-amido synthetase) protein in the HO24/HO comparison that was not observed in the WT seedlings (Figure S2). This protein catalyzes the conjugation of JA to the amino acid isoleucine (JA-Ile), the bioactive form of JA recognized by COI1-JAZ (coronatine insensitive 1-jasmonate ZIM domain) co-receptor complexes [60]. JA-Ile is often produced rapidly in response to biotic or abiotic stimuli and triggers a cascade of events leading to activation of DNA-bound transcription factors [61]. In addition, after hypoxia treatment, the level of chitinases (AAA18586.1) increased in WT24/WT plants, but did not show any change in HO. Despite the absence of any fungi (the seeds used to grow plants were sterilized before use), the response normally connected with biotic factors was apparently triggered by the reduced oxygen content in the WT seedlings. It is possible that the signal pathways associated with defense against pathogens, as well as abiotic factors, are all related to NO, ROS, and hormones. The HO seedlings have a lower amount of ROS; simultaneously, there was a lower response of an antioxidant system. Proteins connected with response to fungal infection, such as chitinases, jasmonate induced protein, or pathogenesis-related proteins, are present in all clusters

In the stress response group, proteins were separated into four clusters (Figure S3). Cluster 1 contained proteins with lower abundance in both genotypes after hypoxia treatment (WT24/WT and HO24/HO). Cluster 2 was comprised of DAP, the abundance of which was mostly higher in HO24/HO. The exception here was wound-responsive family protein (BAK03626.1); the amount of this protein was higher after hypoxia in both genotypes. Cluster 3 was comprised of proteins that decreased in abundance in HO24/HO and HO24/WT24 or in one of comparisons. Cluster 4 was the most heterogeneous and contained proteins that increased in abundance in both HO/WT and WT24/WT or in one of comparisons and others. Barley plants with overexpression of Pgb are more resistant to drought stress [26]. The higher abundance of dehydrin proteins and aquaporins, observed in HO24/HO (Figure S3 cluster 2), was consistent with that.

In the detoxification group, DAP were separated into three clusters (Figure S4). Cluster 1 contained two chloroplast superoxide dismutase (higher abundance in HO/WT and HO24/WT24) and DAP with lower abundance in HO24/HO. Cluster 2 contained proteins with higher abundance in HO24/HO or lower abundance in either HO/WT or WT24/WT. Cluster 3 contained proteins that were less abundant in HO/WT and HO24/WT24, or only in HO24/WT24. This group was mostly peroxidases (nonspecific) (Figure S4). Furthermore, we observed lower glutathione content (both oxidized and reduced form) in seedlings after hypoxia, but there was no difference between genotypes (Figure S1B) indicating that the level of glutathione was not affected by changes in the levels of ROS and NO. Of the remaining statistically significant changes in metabolites also shikimic acid, uric acid, and gamma-aminobutyric acid (GABA) increased in abundance after hypoxia (Figure 1B and Figure 12).

## 3. Discussion

Our findings confirm that barley seedlings with overexpression of Pgb are more resistant to hypoxia, and this higher resistance is due to changes in proteome and metabolome, which suggest a connection with the utilization of alternative energy pathways. Plants can use alternative pathways associated with carbohydrate metabolism, which help them in stressful conditions including hypoxia. Alternative enzymes in the glycolytic pathway can be used in reactions at the level of sucrose, fructose-6-phosphate, glyceraldehyde-3-phosphate, and phosphoenolpyruvate metabolism [62]. These reactions use inorganic pyrophosphate and allow plants to conserve ATP. We found only two of these enzymes, malate synthase (BAJ8837.1) and phosphoenolpyruvate carboxylase (BAJ93490.1), that were downregulated in HO24/HO and one enzyme, 6-phosphofructo-2-kinase/fructose-2,6-bisphosphatase-like (BAK04290.1), involved in sugar metabolism was upregulated in this comparison (Figure 12). However, we also observed the upregulation of Ala and downregulation of Asp in hypoxia-treated seedlings (Figure 12).

The flexibility of metabolism also depends on reaction where pyruvate is transaminated by glutamate to form alanine and 2-oxoglutarate, however, to form glutamate, aspartate aminotransferase uses Asp as a substrate. Then 2-oxoglutarate is used as a substrate of the TCA cycle. The NAD that is required for this reaction is provided by the TCA-cycle enzyme malate dehydrogenase that catalyzes the reaction from oxaloacetate to malate in hypoxic conditions [63]. In addition, we observed changes in pyruvate, L-lactic acid, and 3-phosphoglyceric acid, between HO24 and WT24 (increased abundance in HO24), which could indicate hypoxia-related changes in the carbohydrate metabolism between these two genotypes (Figure 12).

GABA increased under hypoxia treatment in both genotypes (Figure S1). This non-protein amino acid can function as an alternative substrate in the TCA cycle through its conversion to succinate, and thus help during recovery from stress [64]. GABA can be synthesized in at least two ways, through decarboxylation of glutamate (Glu) by glutamate decarboxylase and through degradation of polyamines (PAs) by polyamine oxidase (PAO) and diamine oxidase (DAO). PAs also play an important role in modulating the defense response of plants to diverse environmental stresses [53] and could be the source of NO through an oxidative pathway [54]. We observed a lower abundance of Glu during hypoxia treatment for both genotypes (Figure 12). The abundance of PAO (BAJ99865.1 and CAC42119.1) was lower in WT24/WT, but higher in HO24/WT24. At the same time, the amount of putrescine was lower in HO24 and HO as compared with WT and WT24 (Figure 12). These changes could be due to the fact that GABA arises as a result of spermidine degradation by PAO, and the enzyme itself also takes part in the degradation of other polyamines. In addition, the exact manner in which NO can be produced as a result of the degradation of polyamines is not yet known. However, results for melon subjected to hypoxia indicated the probability of a negative feedback mechanism [65]. GABA can inhibit its own formation from the PA. At the same time, the results point to the extremely important role of polyamines and GABA in response to hypoxia in barley. Pathways connecting the GABA shunt, polyamines, and biosynthesis of ethylene are shown in Figure 12 (all data used in Figure 12 is available in Supplementary Table S5).

In summary, our findings confirm that barley seedlings with overexpression of Pgb are more resistant to hypoxia, and this higher resistance is due to changes in proteome and metabolome. The main changes took place in Ca^2+^ signal transduction, vesicle trafficking protein degradation, and proteins related to disease/defense. GABA shunt can play an extremely important role in hypoxia response/survival and also its relationship with polyamine pathway and ethylene biosynthesis. Several studies have shown that plant phytoglobin overexpression has a positive effect on plant performance under hypoxia [66]. In *Arabidopsis*, the involvement of phytoglobins in ethylene priming to hypoxia suggests that this is because phytoglobin overexpression mimics ethylene priming by removing NO, and thereby activating ERFVII transcription factors that are regulated by degradation through the N-end rule pathway in the presence of both O2 and NO in cells [29]. We found that many of the proteins and other enzymes that assist barley plants in their response to hypoxia were not activated by phytoglobin overexpression. In some cases, such as for regulation of histone isoform abundancy, phytoglobin overexpression actually had an opposite effect to that of hypoxia. This suggests that phytoglobin control of transcriptionally regulated responses to hypoxia is not only controlled by activation of the N-end rule pathway by NO scavenging but also through other, as yet unknown, signaling pathways.

## 4. Materials and Methods

### 4.1. Plant Material

Two barley genotypes were used in the experiment. Wild type (WT) of *Hordeum vulgare* L. cv. Golden Promise was used as a control and transgenic barley lines of the same cultivar overexpressing cDNA of the barley phytoglobin (formerly hemoglobin) gene *HvPGB1-1* (accession number: U94968) controlled by the maize ubiquitin2 promoter. The transgenic line was described in [28] and is designated as HO (for hemoglobin overexpressor).

Barley plants were grown from seeds on moistened filter paper for 8 days in a growth chamber (19 ± 1 °C) with photoperiod of 16 h light/8 h darkness with ~120 μmol·m^−2^·s^−1^ (6480 lux) of light intensity and relative humidity of 70%.

### 4.2. Hypoxia Treatment

The 8-day-old seedlings were placed in a vacuum-sealed container for 24 h. The opaque container was flushed with a gas mixture of 0.1% (*v/v*) O_2_ and 99.9% N_2_ for 20 min to remove all oxygen from the container, after which it was filled with the same gas mixture. Normoxia plants were treated under the same conditions but in a normal atmosphere. After 24 h, the leaves from both types of plants (normoxia and hypoxia conditions, referred to as WT or HO for normoxia, and WT24 or HO24 for hypoxia) were cut and immediately frozen in liquid nitrogen and stored at −80 °C.

### 4.3. Physiological Measurements

All physiological measurements were done at two time points, after 24 h hypoxia treatment and 72 h after treatment was finished (recovery period), using 8 to 10 replicates for each factor. Chlorophyll content was measured using a chlorophyll meter (SPAD-502, Konica Minolta Sensing Inc., Osaka, Japan). The results from SPAD were compared to a standard curve that was obtained by measuring 4 leaves with chlorophyll meter, then, the same leaves were used for chlorophyll isolation. Leaves were ground in a mortar with 96% ethanol. Extract was centrifuged at 11,000 g for 15 min at 4 °C and the supernatant was measured in a UV-Vis spectrophotometer (UV 2700; Shimadzu Corp., Kioto, Japan). Chlorophyll a + b was calculated according to [67] and expressed as mg g^−1^·FW.

Maximum quantum efficiency of PSII (Fv/Fm) was measured on leaves after a dark-adaptation period of 30 min by using leaf-clips and MINI-PAM-II (Heinz Walz GmbH, Effeltrich, Germany).

For fresh and dry weight, shoots were cut, weighed, and oven-dried at 95 °C for 24 h and weighed once more.

### 4.4. Proteomics

#### 4.4.1. Protein Extraction and Digestion

Frozen leaves (0.45 g) were ground in liquid nitrogen, and proteins were extracted using extraction buffer: 2% (*w/v*) sodium deoxycholate (SDC), 10 mM dithiothreitol (DTT), polyvinyl polypyrrolidone (PVPP), 0.1 M triethylammonium bicarbonate (TEAB, pH 8.5), protease inhibitors (Complete™, EDTA free protease inhibitor cocktail, Roche, Basel, Switzerland), and phosphatase inhibitors (PhosSTOP™, Roche, Basel, Switzerland). The homogenate was incubated at 80 °C for 10 min and, then, sonicated in an ice bath for 2 × 15 s with a 30 s break. Samples were vortexed vigorously at room temperature for 30 min and, then, centrifuged for 15 min with 10,000 g and 15 min with 20,000 g. The amount of protein in the supernatant was quantified by amino acid analysis method [68]. Samples were prepared in three biological repetitions and four conditions (two genotype types, WT and HO, and normoxia and hypoxia treatments).

Protein digestion was carried out using the SDC-FASP protocol, as described in [69]. A total of 100 µg protein was mixed with 200 µL 1% (*w/v*) SDC solution in 0.1 M TEAB, pH 8.5, and dialyzed in Microcon spin filter (10,000 g for 15 min at room temperature). Alkylation of free cysteines was performed on the filter membrane using 100 µL 0.05 M iodoacetamide in 1% SDC, 0.1 M TEAB solution, incubated for 30 min in the dark at room temperature. To remove iodoacetamide, samples were centrifuged at 10,000 g for 15 min and washed twice with 1% SDC solution. Digestion of proteins was carried out in 50 µL of trypsin solution in 1% SDC, 0.1 M TEAB, pH 8.5, with an enzyme/protein ratio of 1:50 (*w/w*). Samples were incubated for 6 h, at 37 °C. The peptides were collected by centrifugation and the filter was additionally washed with 1% SDC solution and combined with the peptide solution. SDC was removed from the samples by ethyl acetate extraction from the acidified solution using the phase transfer method [70].

#### 4.4.2. TMT Labeling

Peptide samples were dried using a vacuum centrifuge and dissolved in 50 µL of 0.2 M TEAB. The pH of the solution was checked and adjusted to 8.0. The peptide concentration was determined by Qubit Protein Assay Kit (Thermo Fisher Scientific, Waltham, MA, USA). Peptides (20 µg) were labeled with TMT 10 plex, using 8 tags (127C, 127N, 128C, 128N, 129C, 129N, 130C, and 130N) according to the manufacturer’s protocol (Thermo Fisher Scientific, Waltham, MA, USA). Three replicates of the above four treatments were labeled with three series of the TMT tags: 127C, 127N, 128C, and 128N for replicate one; 129C, 129N, 130C, and 130N for replicate two; 127C, 127N, 128C, and 128N for replicate three of each treatment. After labeling, replicates one and two and replicates two and three were mixed into two sets of TMT labels and desalted with Poros^®^20 R2 reversed phase microcolumns.

#### 4.4.3. LC-MS/MS Analysis

TMT labeled samples were analyzed by 2D-LC-MS, using high pH reverse phase (RP) fractionation as a first dimension followed by low pH RP chromatography, coupled to MS. For high pH, RP fractionation samples were separated on an Ultimate3000 HPLC system (ThermoScientific, Waltham, MA, USA) using ACQUITY CSH C18 1.7 µm column (300 µm × 100 mm) (Waters S.A.S. Saint-Quentin, France) with a linear gradient from 2% to 60% of buffer B in 1 h. Buffer A was H_2_O, 20 mM ammonium formate, pH 9; buffer B was 80% acetonitrile (ACN) and 20% buffer A. Ten subfractions were collected (6 min per fraction) and combined into 5 fractions; thus, fraction 1 contained subfractions 1 and 6. The fractions were dried out in a speed-vac and resolubilized in 2% ACN, 98% H_2_O, 0.1% trifluoroacetic acid (TFA).

Full fraction volume after resolubilization was injected on the second dimension LC-MS on a Ultimate3000 RSLCnano HPLC system connected to a QExactive HF MS system (ThermoScientific, Waltham, MA, USA). Samples were loaded on a cartridge precolumn PepMap-100 C18, 5*0.3 mm (ThermoScientific, Waltham, MA, USA) in 2% ACN, 98% H_2_O, 0.1% TFA, at 3 mL/min and, then, separated on an EASY-Spray™ PepMap RSLC C18 2µm column (50 cm 75 µm^−1^, Thermo Scientific, Waltham, MA, USA). Separation was performed with a gradient of ACN, 0.1% FA (buffer B) in H_2_O, 0.1% FA (buffer A) from 4% to 32% buffer B in 2 h at 0.27 µL/min, at 25 °C.

MS analysis was carried out in a DDA mode with 1 MS1 scan, followed by TOP20 MS2 scans. MS1 parameters were 120,000 resolution, 3e6 AGC target, maximum IT 100 ms, and scan range 300–1600 m/z. MS2 parameters were 60,000 resolution, 1e5 AGC target, maximum IT 100 ms, isolation window 1.4 m/z, isolation offset 0.2 m/z, fixed first mass 110 m/z, (N)CE 34, minimum AGC 1e3, exclude unassigned and 1, 6 to 8 charges, preferred peptide match, exclude isotopes ON, dynamic exclusion 30 s.

#### 4.4.4. Protein Identification and Quantification

Raw MS/MS data were processed using Proteome Discoverer 2.1 (Thermo Scientific, Waltham, MA, USA) and searched using the Mascot search engine. The two fractionated TMT sets were searched through the processing workflow in batch mode, followed by a multiconsensus workflow combining the files. The Mascot parameters for protein identification were defined as follows: database NCBI [71], *Hordeum vulgare* protein database (updated on 18 October 2018); precursor mass tolerance 20 ppm; fragment mass tolerance 0.05 Da; digestion, trypsin with two missed cleavages allowed; fixed modification, carbamidomethyl (C), TMT6plex (K), and TMT6plex (N-term); variable modification, methionine oxidation (M). The Percolator was used for peptide validation. Protein quantification was performed using the reporter ion quantifier node (processing workflow) to estimate the peak intensity of the reporter ions, followed by the use of the peptide and protein quantifier (consensus workflow) node embedded in Proteome Discoverer 2.1, where protein abundance is calculated as the average of the three most abundant distinct peptides identified for the protein. A cut-off value of at least one unique peptide per protein was applied. The list of all identified and quantified proteins can be found in Table S1. The mass spectrometry proteomics data have been deposited in the ProteomeXchange Consortium via the PRIDE [72] partner repository with the dataset identifier PXD017103.

#### 4.4.5. Measurement of Hydrogen Peroxide (H_2_O_2_) Content

The concentration of H_2_O_2_ in barley seedlings was determined, according to [73]. Freshly collected leaves (100 mg) were immediately homogenized in Eppendorf tubes with ceramic beads by Fastprep grinder in cooled 0.1% (*w/v*) trichloroacetic acid. After centrifugation at 15,000 g for 15 min at 4 °C, the supernatant was collected for further analysis. A reaction mixture was prepared (0.125 mL supernatant, 0.5 mL freshly prepared 1 M KI in 10 mM potassium phosphate, pH 7.0, and 0.25 mL 10 mM potassium phosphate, pH 7.0) and incubated for 10 min. Then 0.2 mL was taken for the measurement at 390 nm in the plate reader. As a blank, the reaction mixture without plant extract was used and the standard curve was prepared using 0.88 µM H_2_O_2_ (Sigma-Aldrich, Saint Louis, MO, USA). Measurements of H_2_O_2_ concentration were done in four independent biological replicates and expressed as µmol·g^−1^·FW.

#### 4.4.6. Measurement of Oxidation of Epinephrine by Superoxide Anions (O_2_^•−^)

Measurement of O_2_^•−^ generation was done according to [74]. Barley leaves (100 mg) were homogenized in 0.05 M Tris-HCl, pH 7.5, and centrifuged at 12,000 g for 15 min at 4 °C. The supernatant was collected for further analyses. The oxidation of epinephrine to adrenochrome was measured in the reaction mixture (0.05 mL of supernatant, 0.05 mL 0.05 M Tris-HCl, pH 7.5, 0.05 mL of 60 mM epinephrine prepared in 50 mM HCl) at 480 nm (microplate reader) for 5 min. Measurements were done in three independent biological replicates The epinephrine extinction coefficient was ε = 4.02 mM^−1^·cm^−1^ and expressed as µmol·min^−1^·g^−1^·FW.

### 4.5. Metabolomics

#### 4.5.1. Metabolite Extraction

Total metabolites were extracted from 50 mg fresh weight by 1 mL methanol/acetonitrile/water (4:4:2 *v/v/v*) chilled to −20 °C (spiked with 13C6 sorbitol and reserpine in a concentration of 0.4 mg·L^−1^, as internal standards). Samples were placed in an ultrasonic bath (2 min, 4 °C) followed by shaking (15 min, 4 °C) and centrifuged (16,000 g for 5 min). The supernatants were split in fractions for GC-MS, quality control (QC) for GC-MS and LC-MS in a 6:1:12 ratio.

#### 4.5.2. GC-MS Analysis and Data Extraction

The GC-MS QC samples were pooled and split into 6 samples before all samples were lyophilized. All samples for GC-MS were derivatized and analyzed as in [75]. QC samples for correction of signal drift were injected after every 6th sample. Deconvolution and annotation were done in Masshunter Unknown analysis version B.08.00 (Agilent Technologies, Santa Clara, CA, USA). Compounds were considered as putatively annotated (MSI level 2, after [76]) by matching the deconvoluted mass spectra against the FiehnLib [77] with a minimum match factor 70. Annotation was further supported by the use of retention indices, which was generated using fatty acid methyl esters (FAME mix). Peak alignment and quantification were conducted in Masshunter Quantitative Analysis version B.08.00 (Agilent Technologies, Santa Clara, CA, USA).

#### 4.5.3. LC-MS Analysis and Data Extraction

Samples were resuspended in 30 µl 1% formic acid (FA) in water before centrifugation at 16,000 g for 5 min and transfer of 25 µL to HPLC vial containing 100 µL inserts. Then, 3 µl of each sample was transferred to a new vial and used for QC and annotation runs. 3 µL of the samples were injected on an Agilent 1290 Ininity HPLC system (Agilent Technologies, Santa Clara, CA, USA) equipped with an Agilent Zorbax Eclipse Plus C18 column (2.1 × 150 mm, 1.8 μm) with a 50 mm guard-column, both kept at 40 °C. The chromatographic gradient was run at a flow rate of 400 μL/min with the following solvent composition of A (0.1% FA, water) and B (0.1% FA, acetonitrile): 97% A from 0 to 1.5 min, 97% to 60% A from 1.5 to 4.5 min and 60% to 5% A from 4.5 to 7.5 min, 5% A from 7.5 to 10.1 min, 5% to 97% A from 10.1 to 10.5 min before equilibration for 3.5 min with the initial conditions. Eluting compounds were detected by a 6530B quadrupole time of flight mass spectrometer (Agilent Technologies, Santa Clara, CA, USA) operated in both negative and positive ion mode scanning from 40 to 1050 m/z with the following settings: 3 scans/s, gas temp at 325 °C, drying gas at 8 L/min, nebulizer at 35 psig, sheath gas temp at 350 °C, sheath gas flow at 11 min/L, VCap at 3500 V, fragmentor at 125 V, and skimmer at 65 V. Each spectrum was internally calibrated during analysis using the signals of purine and hexakis(1H,1H,3H-tetrafluoropropoxy)phosphazine, which was delivered to a second needle in the ion source by an isocratic pump running with a flow of 20 µL/min. The QC sample was not only used for quality control but also for fragmentation analysis, and thus compound annotation. This sample was analyzed in “all-ion” mode using 0, 10, 20, and 40 V in collision energy (CE) and, moreover, analyzed in data-dependent analysis MS/MS mode fragmenting ions (using 10 and 40 V CE). QC samples for correction of signal drift were run in MS-mode and injected after every 6th sample.

The raw data were converted to mzXML format using MSConvert [78] and processed using MZmine 2 [79] with the chromatogram deconvolution module Wavelets ADAP [80]. Compounds were putatively annotated to MSI level 3 [76] in Masshunter Qualitative Analysis B.08.00 (Agilent Technologies, Santa Clara, CA, USA) using the files acquired in “all-ion” and MS/MS mode matching against the Agilent METLIN Metabolomics Database and Library (Agilent Technologies, Santa Clara, CA, USA).

#### 4.5.4. General Data Filtering and Processing

Compounds not present in 75% of the QC samples, with a coefficient of variation (CV) of >30% in the QC samples or with an average abundance of less than 5 times as compared with the blank extraction samples, were excluded. The compound list was uploaded to MetaboAnalyst.ca [81] where features with >50% missing values were removed and remaining missing values were estimated using K-nearest neighbor (KNN). The signal was normalized by using the QC samples. Compound lists from LC-MS and GC-MS were merged prior to log2 transformation and auto scaling in MetaboAnalyst [81].

### 4.6. Statistical Analyses

Identified proteins from Proteome Discover 2.1 were analyzed using LimmaRP [82]. For differentially abundant proteins (DAP), a *q*-value below 0.05 was considered statistically significant for two-group comparisons. For metabolomic analyses, MetaboAnalyst 4.0 [81] was used, with one-way ANOVA using a cutoff at 0.05 and Tukey’s HSD post-hoc analysis. To visualize the differences between all, Venn diagram and heatmaps were constructed using R version 3.5.1 “Feather Spray” and packages VennDiagram [83], ComplexHeatmap [84]. For physiological parameters, a one-way ANOVA was used with Tukey’s HSD post-hoc analysis.

## Supplementary Materials

Supplementary materials can be found at https://www.mdpi.com/1422-0067/21/4/1546/s1. Figure S1: Box and whisker plots for selected metabolites that showed statistically significant differences between the treatments with *p* < 0.5 ANOVA with Tukey’s HSD. (A) Diagrams for amino acids; (B) other metabolites; Figure S2: Heatmap displaying DAP with function related to defense. Seedling of wild type shown as WT and with overexpression of phytoglobin as HO, seedlings after hypoxia treatment shown as WT24 (wild type) and HO24 (overexpression of phytoglobin). The color scale illustrates the relative abundance level of each protein across the 3 biological samples; red and blue indicate higher and lower expression in comparisons. Heatmap is showing 4 largest clusters created with k-means. Asterisk indicate level of *p* (* *p* < 0.05; ** *p* < 0.01; *** *p* < 0.001); Figure S3: Heatmap displaying DAP with function related to stress response. Seedling of wild type shown as WT and with overexpression of phytoglobin as HO, seedlings after hypoxia treatment shown as WT24 (wild type) and HO24 (overexpression of phytoglobin). The color scale illustrates the relative abundance level of each protein across the 3 biological samples; red and blue indicate higher and lower expression in comparisons. Heatmap is showing 4 largest clusters created with k-means. Asterisk indicate level of *p* (* *p* < 0.05; ** *p* < 0.01; *** *p* < 0.001); Figure S4: Heatmap displaying DAP with function related to detoxification. Seedling of wild type shown as WT and with overexpression of phytoglobin as HO, seedlings after hypoxia treatment shown as WT24 (wild type) and HO24 (overexpression of phytoglobin). The color scale illustrates the relative abundance level of each protein across the 3 biological samples; red and blue indicate higher and lower expression in comparisons. Heatmap is showing 3 biggest clusters created with k-means. Asterisk indicate level of *p* (* *p* < 0.05; ** *p* < 0.01; *** *p*< 0.001); Figure S5: Histones protein sequences with possible post-translational modification sides of acetylation (lysines—K, blue), methylation (lysines—K or arginines—R, green) or phosphorylation (serines—S or threonines—T, yellow). The possible modified amino acids were indicated according to [56,85,86,87]; Table S1: All proteins identified and quantified from Proteome Discoverer; Table S2: All differenced abundant proteins; Table S3: All metabolites identified and quantified; Table S4: Selected metabolites; Table S5: Data for proteins and metabolites in Figure 12; Table S6: Data of identified peptides for histone proteins.
