# Supplementary material for: The Hypoxic Proteome and Metabolome of Barley (Hordeum vulgare L.) with and without Phytoglobin Priming
Source: Int J Mol Sci. 2020 Feb 24;21(4):1546. doi: 10.3390/ijms21041546 (PMC7073221; doi:10.3390/ijms21041546)
Supplement: Supplementary file 1 [file ijms-21-01546-s001.zip › ijms-726488-SI-to conversion/Figure S2_defense.pdf]

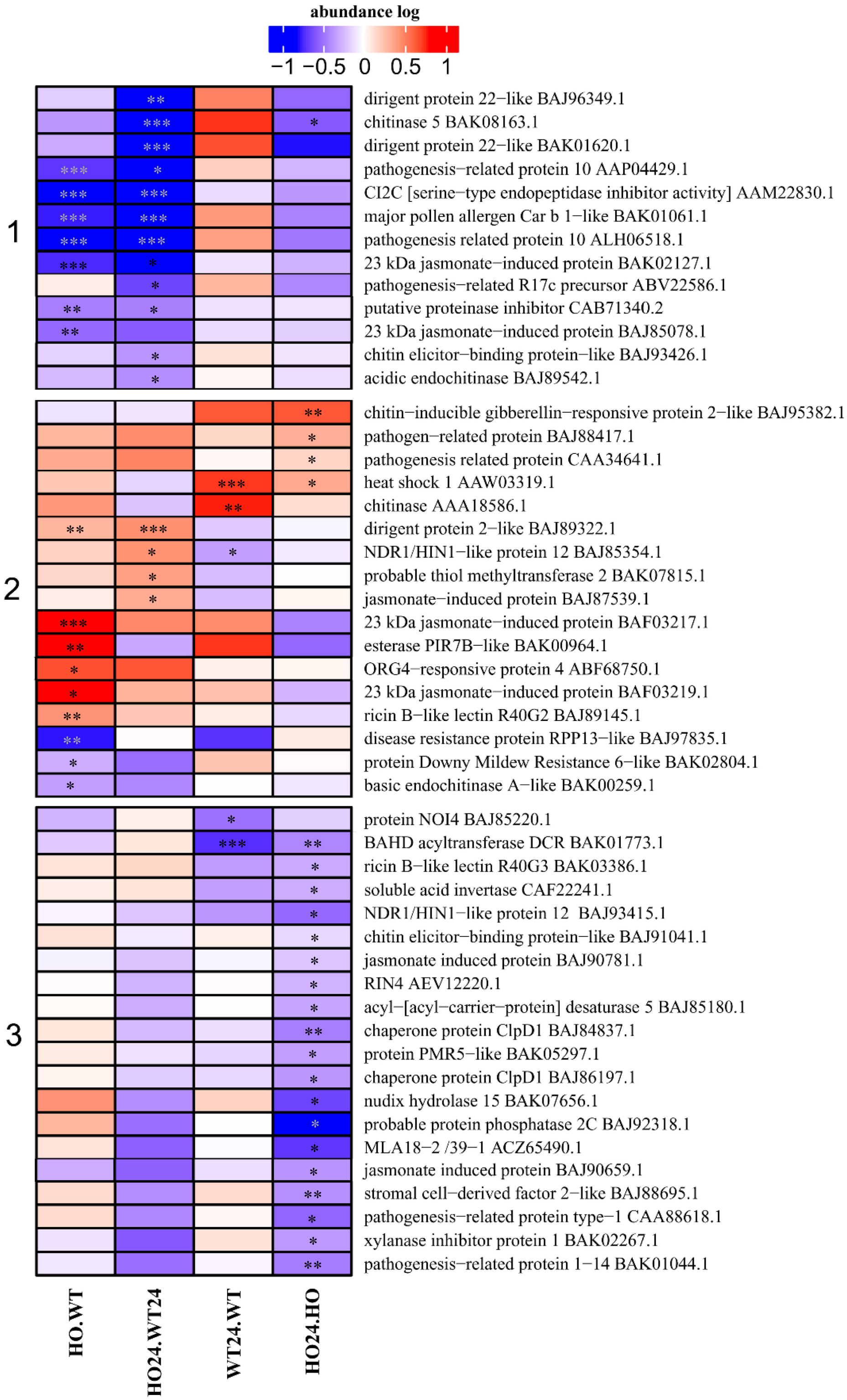

abundance log
−1 −0.5 0 0.5 1
1
2
3
dirigent protein 22−like BAJ96349.1
chitinase 5 BAK08163.1
dirigent protein 22−like BAK01620.1
pathogenesis−related protein 10 AAP04429.1
CI2C [serine−type endopeptidase inhibitor activity] AAM22830.1
major pollen allergen Car b 1−like BAK01061.1
pathogenesis related protein 10 ALH06518.1
23 kDa jasmonate−induced protein BAK02127.1
pathogenesis−related R17c precursor ABV22586.1
putative proteinase inhibitor CAB71340.2
23 kDa jasmonate−induced protein BAJ85078.1
chitin elicitor−binding protein−like BAJ93426.1
acidic endochitinase BAJ89542.1
chitin−inducible gibberellin−responsive protein 2−like BAJ95382.1
pathogen−related protein BAJ88417.1
pathogenesis related protein CAA34641.1
heat shock 1 AAW03319.1
chitinase AAA18586.1
dirigent protein 2−like BAJ89322.1
NDR1/HIN1−like protein 12 BAJ85354.1
probable thiol methyltransferase 2 BAK07815.1
jasmonate−induced protein BAJ87539.1
23 kDa jasmonate−induced protein BAF03217.1
esterase PIR7B−like BAK00964.1
ORG4−responsive protein 4 ABF68750.1
23 kDa jasmonate−induced protein BAF03219.1
ricin B−like lectin R40G2 BAJ89145.1
disease resistance protein RPP13−like BAJ97835.1
protein Downy Mildew Resistance 6−like BAK02804.1
basic endochitinase A−like BAK00259.1
protein NOI4 BAJ85220.1
BAHD acyltransferase DCR BAK01773.1
ricin B−like lectin R40G3 BAK03386.1
soluble acid invertase CAF22241.1
NDR1/HIN1−like protein 12 BAJ93415.1
chitin elicitor−binding protein−like BAJ91041.1
jasmonate induced protein BAJ90781.1
RIN4 AEV12220.1
acyl−[acyl−carrier−protein] desaturase 5 BAJ85180.1
chaperone protein ClpD1 BAJ84837.1
protein PMR5−like BAK05297.1
chaperone protein ClpD1 BAJ86197.1
nudix hydrolase 15 BAK07656.1
probable protein phosphatase 2C BAJ92318.1
MLA18−2 /39−1 ACZ65490.1
jasmonate induced protein BAJ90659.1
stromal cell−derived factor 2−like BAJ88695.1
pathogenesis−related protein type−1 CAA88618.1
xylanase inhibitor protein 1 BAK02267.1
pathogenesis−related protein 1−14 BAK01044.1
HO.WT
HO24.WT24
WT24.WT
HO24.HO
