# Supplementary material for: The Hypoxic Proteome and Metabolome of Barley (Hordeum vulgare L.) with and without Phytoglobin Priming
Source: Int J Mol Sci. 2020 Feb 24;21(4):1546. doi: 10.3390/ijms21041546 (PMC7073221; doi:10.3390/ijms21041546)
Supplement: Supplementary file 1 [file ijms-21-01546-s001.zip › ijms-726488-SI-to conversion/Figure S3_stress.pdf]

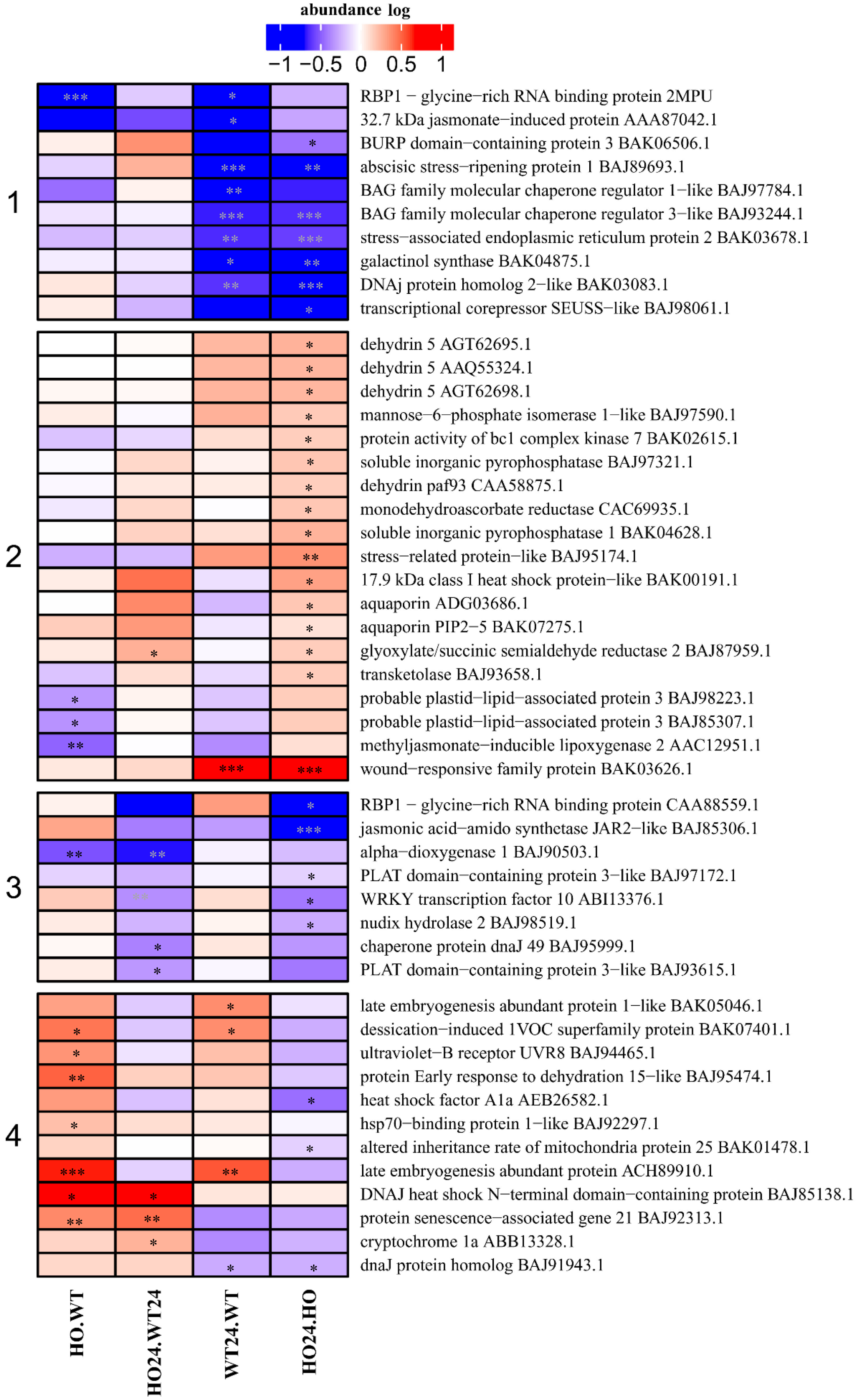

abundance log
−1 −0.5 0 0.5 1
1
RBP1 − glycine−rich RNA binding protein 2MPU
32.7 kDa jasmonate−induced protein AAA87042.1
BURP domain−containing protein 3 BAK06506.1
abscisic stress−ripening protein 1 BAJ89693.1
BAG family molecular chaperone regulator 1−like BAJ97784.1
BAG family molecular chaperone regulator 3−like BAJ93244.1
stress−associated endoplasmic reticulum protein 2 BAK03678.1
galactinol synthase BAK04875.1
DNAj protein homolog 2−like BAK03083.1
transcriptional corepressor SEUSS−like BAJ98061.1
2
dehydrin 5 AGT62695.1
dehydrin 5 AAQ55324.1
dehydrin 5 AGT62698.1
mannose−6−phosphate isomerase 1−like BAJ97590.1
protein activity of bc1 complex kinase 7 BAK02615.1
soluble inorganic pyrophosphatase BAJ97321.1
dehydrin paf93 CAA58875.1
monodehydroascorbate reductase CAC69935.1
soluble inorganic pyrophosphatase 1 BAK04628.1
stress−related protein−like BAJ95174.1
17.9 kDa class I heat shock protein−like BAK00191.1
aquaporin ADG03686.1
aquaporin PIP2−5 BAK07275.1
glyoxylate/succinic semialdehyde reductase 2 BAJ87959.1
transketolase BAJ93658.1
probable plastid−lipid−associated protein 3 BAJ98223.1
probable plastid−lipid−associated protein 3 BAJ85307.1
methyljasmonate−inducible lipoxygenase 2 AAC12951.1
wound−responsive family protein BAK03626.1
3
RBP1 − glycine−rich RNA binding protein CAA88559.1
jasmonic acid−amido synthetase JAR2−like BAJ85306.1
alpha−dioxygenase 1 BAJ90503.1
PLAT domain−containing protein 3−like BAJ97172.1
WRKY transcription factor 10 ABI13376.1
nudix hydrolase 2 BAJ98519.1
chaperone protein dnaJ 49 BAJ95999.1
PLAT domain−containing protein 3−like BAJ93615.1
4
late embryogenesis abundant protein 1−like BAK05046.1
dessication−induced 1VOC superfamily protein BAK07401.1
ultraviolet−B receptor UVR8 BAJ94465.1
protein Early response to dehydration 15−like BAJ95474.1
heat shock factor A1a AEB26582.1
hsp70−binding protein 1−like BAJ92297.1
altered inheritance rate of mitochondria protein 25 BAK01478.1
late embryogenesis abundant protein ACH89910.1
DNAJ heat shock N−terminal domain−containing protein BAJ85138.1
protein senescence−associated gene 21 BAJ92313.1
cryptochrome 1a ABB13328.1
dnaJ protein homolog BAJ91943.1
HO.WT
HO24.WT24
WT24.WT
HO24.HO
