# Supplementary material for: The Hypoxic Proteome and Metabolome of Barley (Hordeum vulgare L.) with and without Phytoglobin Priming
Source: Int J Mol Sci. 2020 Feb 24;21(4):1546. doi: 10.3390/ijms21041546 (PMC7073221; doi:10.3390/ijms21041546)
Supplement: Supplementary file 1 [file ijms-21-01546-s001.zip › ijms-726488-SI-to conversion/Figure S4_detox.pdf]

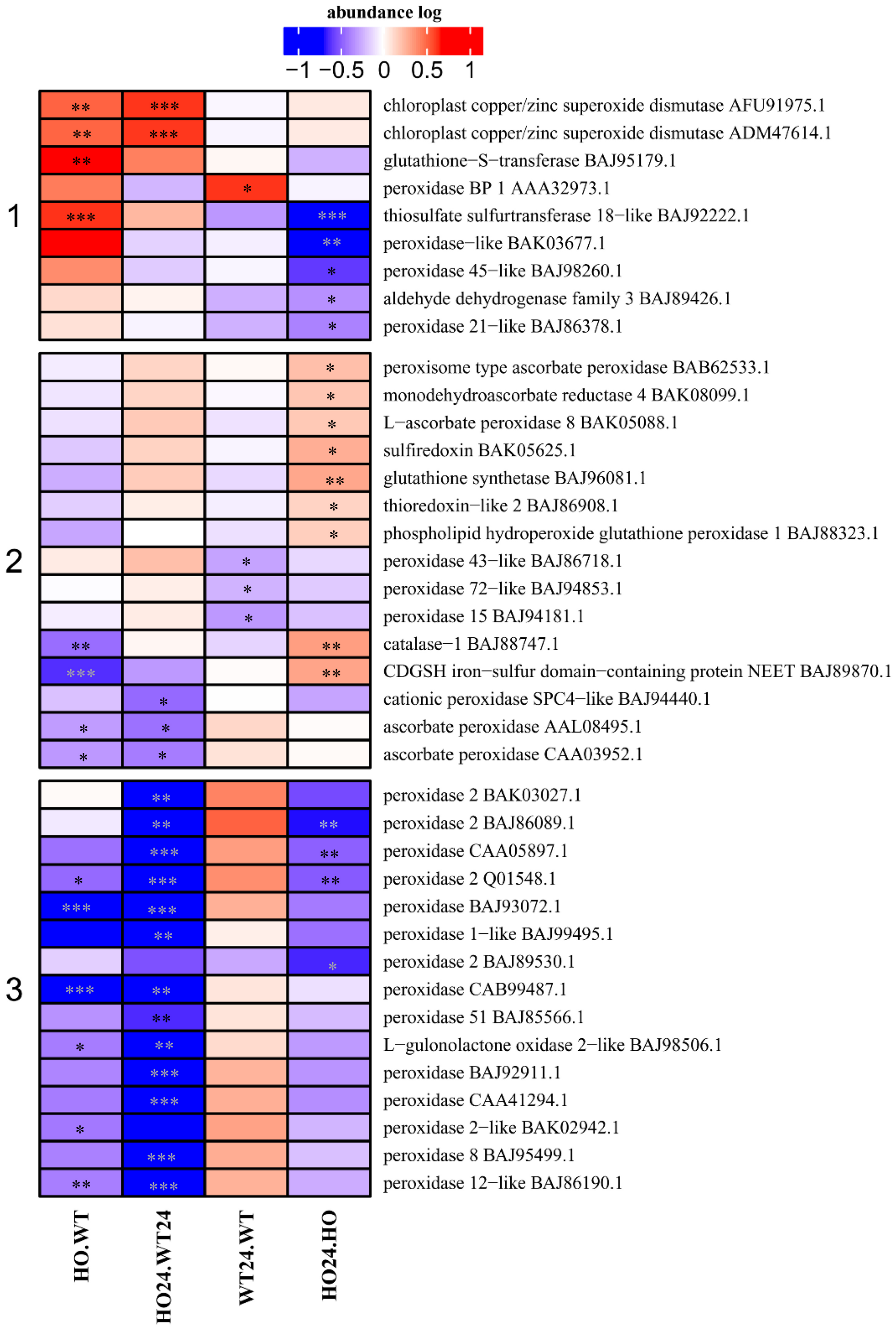

abundance log
−1 −0.5 0 0.5 1
1
2
3
chloroplast copper/zinc superoxide dismutase AFU91975.1
chloroplast copper/zinc superoxide dismutase ADM47614.1
glutathione−S−transferase BAJ95179.1
peroxidase BP 1 AAA32973.1
thiosulfate sulfurtransferase 18−like BAJ92222.1
peroxidase−like BAK03677.1
peroxidase 45−like BAJ98260.1
aldehyde dehydrogenase family 3 BAJ89426.1
peroxidase 21−like BAJ86378.1
peroxisome type ascorbate peroxidase BAB62533.1
monodehydroascorbate reductase 4 BAK08099.1
L−ascorbate peroxidase 8 BAK05088.1
sulfiredoxin BAK05625.1
glutathione synthetase BAJ96081.1
thioredoxin−like 2 BAJ86908.1
phospholipid hydroperoxide glutathione peroxidase 1 BAJ88323.1
peroxidase 43−like BAJ86718.1
peroxidase 72−like BAJ94853.1
peroxidase 15 BAJ94181.1
catalase−1 BAJ88747.1
CDGSH iron−sulfur domain−containing protein NEET BAJ89870.1
cationic peroxidase SPC4−like BAJ94440.1
ascorbate peroxidase AAL08495.1
ascorbate peroxidase CAA03952.1
peroxidase 2 BAK03027.1
peroxidase 2 BAJ86089.1
peroxidase CAA05897.1
peroxidase 2 Q01548.1
peroxidase BAJ93072.1
peroxidase 1−like BAJ99495.1
peroxidase 2 BAJ89530.1
peroxidase CAB99487.1
peroxidase 51 BAJ85566.1
L−gulonolactone oxidase 2−like BAJ98506.1
peroxidase BAJ92911.1
peroxidase CAA41294.1
peroxidase 2−like BAK02942.1
peroxidase 8 BAJ95499.1
peroxidase 12−like BAJ86190.1
HO.WT
HO24.WT24
WT24.WT
HO24.HO
