# Supplementary material for: The Hypoxic Proteome and Metabolome of Barley (Hordeum vulgare L.) with and without Phytoglobin Priming
Source: Int J Mol Sci. 2020 Feb 24;21(4):1546. doi: 10.3390/ijms21041546 (PMC7073221; doi:10.3390/ijms21041546)
Supplement: Supplementary file 1 [file ijms-21-01546-s001.zip › ijms-726488-SI-to conversion/Figure S5_histone modification.docx]

Supplemental Fig S5. Histones protein sequences with possible post-translational modification sides of acetylation (lysines – K, blue), methylation (lysines – K or arginines – R, green) or phosphorylation (serines – S or threonines – T, yellow). The possible modified amino acids were indicated according to Brabencová et al. (2017), Peterson and Laniel (2004), Ageeva-Kieferle et al. (2019), Hartl et al. (2017). Peptides used for protein identification are underlined and the one that specific for individual protein are in bold.

**H2A**

**BAJ84992.1**

1 MAGRKGGDRK KSVTRSVKAG LQFPVGRIGR YLKKGRYAQR VGSGAPVYLA AVLEYLAAEL

61 LELAGNAAKD NKKTRIIPRH LLLAVR**NDQE** **LGK**LLAGVTI AHGGVIPNIN SVLLPKKSPA

121 AAEKEAKSPK KKADTKSPKK KATKE

**BAK07505.1**

1 MAGRKGGERK KAVARSVKAG LQFPVGRIGR YLKKGRYAQR VGSGAPVYLA AVLEYLAAEL

61 LELAGNAAKD NKKTRIIPRH LLLAVRNDQE LGRLLAGVTI AHGGVIPNIN SVLLPKKSPA

121 AAEKEATKSP KK**KAAATK**SP KK**KAAATKE**

**BAK05388.1**

1 MAGRKGGERK KAVTRSVKAG LQFPVGRIGR YLKKGRYAQR VGSGAPVYLA AVLEYLAAEL

61 LELAGNAAKD NKKTRIIPRH LLLAVRNDQE LGRLLAGVTI AHGGVIPNIN SVLLPKKSPA

121 AAEKEATKSP KKKAATKSPK KAAAKV

**BAJ95629.1**

1 MAGRKGGDRK KAVTRSVKAG LQFPVGRIGR YLKKGRYAQR VGSGAPVYLA AVLEYLAAEV

61 LELAGNAAKD NKKTRIIPRH LLLAVRNDQE LGRLLAGVTI AHGGVIPNIN SVLLPK**KSAA**

121 **AAEKEATK**SP KKKAATKSPK KKAPATKE

**BAK00643.1**

1 MAGRKGGDRK KAVTRSVKAG LQFPVGRIGR YLKKGRYAQR VGSGAPVYLA AVLEYLAAEV

61 LELAGNAAKD NKKTRIIPRH LLLAVRNDQE LGRLLAGVTI AHGGVIPNIN SVLLPK**KSAA**

121 **AAEKEATK**SP KKKAATKSPK KKAATKSPKK KTAAKE

**BAK00739.1**

1 MDASATVAAG KGKKGAAGRK AGGPRKKSVS RSVK**AGLQFP** **VSR**IGRFLKK GRYAQRVGSG

61 APVYLAAVLE YLAAELLELA GNAAKDNKKS RITPR**HLLLA** **IR**NDEELGKL LAGITIAHGG

121 VIPNINPVLL PKKTAEKSPK EPKSPKKTAK SPKKA

**BAJ96803.1**

1 MDASGTGAGA KGKKGAAGRK AGGPRKKSVT RSVKAGLQFP VGSIGRYLKK GRYAKRVGTG

61 APVYLAAVLE YLAAELLELA GNAAKDNKKS RIVPRHLLLA VRNDQELGR**L** **LAGVTIAHGG**

121 **VLPNINPVLL** **PK**R**TAEKEPK** EGKSPKKAAK SPKKADKKA

**BAJ86067.1**

1 MDASGTVAKG KKGAAGRKAG GPRKKSVSRS VKAGLQFPVG RIGRYLKKGR YAQRVGTGAP

61 VYLAAVLEYL AAELLELAGN AAKDNKKSRI IPRHLLLAVR NDEELGK**LLA** **GVTIAHGGVV**

121 **PNINTVLLPK** RTAEKEGKAP KSPKKAPKSP KKAAKSPK**KA** **ATPK**KA

**BAK04720.1**

1 MEVSGAAAKG KKGAAGRKAG GPRKKSVTRS VKAGLQFPVG RIGRYLKKGR YAQRVGTGAP

61 VYLAAVLEYL AAELLELAGN AAKDNKKSRI IPRHLLLAVR NDEELGK**LLA GVTIAHGGVI**

121 **PK**INPVLLPK RTAEKEGKGP KSPKKATKSP KKATKA

**H2A.Z-like**

**BAJ87489.1**

1 MAGKGGK**GLL** **AAK**TTAAKGA ADKDKDKKKA PVSRSSRAGI QFPVGRIHRQ LKQRVSANGR

61 VGATAAVYSA AILEYLTAEV LELAGNASKD LKVKRITPR**H** **LQLAIRGDEE** **LDTLIKGTIA**

121 **GGGVIPHIHK** SLINKTSKE

**BAJ94187.1**

1 MTGGKSGGKA SGSKSAQSRS SKAGLAFPVG RVHRLLRKGN YAQRVGAGAP VYLAAVLEYL

61 AAEILELAGN AARDNKKTRI IPR**HLQLAIR** NDEELNKLLG HVTIAQGGVL PNIHQNLLPK

121 KTAKGKNPSQ EL

**BAJ90024.1**

1 MAGR**GKAIGA** **GAVK**KATSRS SK**AGLQFPVG** **R**IARFLKAGK YAERVGAGAP VYLAAVLEYL

61 AAEVLELAGN AARDNKKTRI VPR**HIQLAVR** **NDEELTKLLG** **GATIASGGVM PNIHQHLLPK**

121 KASSSK**ASTV** **DDDDN**

**BAK03725.1**

1 MAGRGK**AIGS** **GAAKK**AISRS SKAGLQFPVG RIARFLKAGK YAERVGAGAP VYLAAVLEYL

61 AAEVLELAGN AARDNKKTRI VPRHIQLAVR NDEELSRLLG MVTIASGGVM PNIHNLLLPK

121 **KAGGSAKAAA** **ADDDS**

**BAK05816.1**

1 MAIAGSGRGK **AKPAASAK**SV SRSSKAGLQF PVGRVARYLK VGKYAERVGA GAPVYLAAVL

61 EYLAAETLEL AGNAARDNKK NRIVPRHIQL AVRNDEELSR LLGSVTIAAG GVLPSIHTTL

121 LPKKAGK**AKG** **DIGSASQEF**

**BAJ95543.1**

1 M**ATAAGGGGR** GKPKGSKSVS RSVKAGLQFP VGRVARHLKV GRYAQRVGAG APVYLCAVLE

61 YLAAEALELA GNAARDNKKT RITPRHIQLA VRNDEELSRL LGGVTIAAGG VLPNINSVLL

121 PKKAGK**AAAG** **TGGSASQSQE** **F**

**H2B**

**BAJ87490.1**

1 MAPK**ADKKPA** **AENKVEK**AAE KTPAGKKPKA EKRLPAGK**TA** **SKEAGGEAK**T RGRKKGSKAK

61 KSVETYKIYI FKVLKQVHPD IGISSKAMSI MNSFINDIFE KLAGESAKLA RYNKKPTITS

121 REIQTSVRLV LPGELAKHAV SEGTKAVTKF TSS

**BAK06939.1**

1 MAPKAEKKPA AEK**KPVEAEK** KPKAEKRVPG KDGGADKKKK KAKKSVETYK IYIFKVLKQV

61 HPDIGISSKA MSIMNSFIND IFEKLAGESA KLARYNKKPT ITSREIQTAV RLVLPGELAK

121 HAVSEGTKAV TKFTSS

**BAK06188.1**

1 MAPKAEK**KPA** **PEKTPAAK**KP AAEKRPAAGK AASKEGGEKK GKKKSKESVE TYKIYIFKVL

61 KQVHPDIGIS SKAMSIMNSF INDIFEKLAG ESAKLARYNK KPTITSREIQ TSVRLVLPGE

121 LAKHAVSEGT KAVTKFTSS

**BAK07641.1**

1 MAPKAEK**KPV** **AEKTPAAEK**T TAGKKPKAEK RPPK**SKEGGE** **K**KGKKKSKKS VETYKIYIFK

61 VLKQVHPDIG ISSKTMSIMN SFINDIFEKL AGESAKLARY NKKPTITSRE IQTSVRLVLP

121 GELAKHAVSE GTKAVTKFTS A

**BAK04637.1**

1 MAPK**ADKKPA** **AESK**VEKAAE KTPAGKKPKA EKRVPAGKTA AKEGEGKTRG RKKGGKAKK**S**

61 **AETYK**LYIFK VLKQVHPDVG ISSKAMSIMN SFINDIFEKL AGESAKLARY NKKPTVTSRE

121 IQTSVRLVLP GELAKHAVSE GTKAVTKFTS A

**BAK06534.1**

1 MAPKAEK**KPV** **AEKAEKSTAG** **KK**TKAEK**RPP** **ASK**EGGEKKG KKKSKKSVET YKIYIFKVLK

61 QVHPDIGISS KAMSIMNSFI NDIFEKLAGE SAKLARYNKK PTITSREIQT SVRLVLPGEL

121 AKHAVSEGTK AVTKFTSA

**BAJ97285.1**

1 MAPKAEK**KPV** **AEKAEK**TTAG KKTKAEK**RPP** **ASK**EGGEKKG KKKSKKSVET YKIYIFKVLK

61 QVHPDIGISS KAMSIMNSFI NDIFEKLAGE SAKLARYNKK PTITSREIQT SVRLVLPGEL

121 AKHAVSEGTK AVTKFTSA

**BAJ96869.1**

1 LCSARLELLI SFRDGRFGPR GPNRPSRSPL SVSLLLLQPT QTSLSPSKKK QQSMAPKAAE

61 KKPVEKSPAG KKPKAEKKVP ASKEGGDKKG KKKSKKSVET YKIYIFKVLK QVHPDIGISS

121 KAMSIMNSFI NDIFEKLAGE SAKLARYNKK PTITSREIQT SVRLVLPGEL AKHAVSEGTK

181 AVTKFTSS

**BAJ94839.1**

1 MAPKAAEKKP VEKTPAGKKP KAEKKVPASK **EGAGGEKK**GK KKSKKSVETY KIYIFKVLKQ

61 VHPDIGISSK AMSIMNSFIN DIFEKLAGES AKLARYNKKP TITSREIQTS VRLVLPGELA

121 KHAVSEGTKA VTKFTSS

**BAJ87396.1**

1 MAPKADKK**AA** **AENKVEK**AAA EKAPAGKKPK AEKRLPAGKT AAK**EGAGGEA** **K**ARGRKKGSK

61 AKKGVETYKI YIFKVLKQVH PDIGISSK**AM** **SIMNSFVNDI** **FEKLAAESAK** LARYNKKPTV

121 TSREIQTSVR LVLPGELAKH AVSEGTKAVT KFTSS

**BAJ88080.1**

1 MAPKAAEKKP VEKTPAGKKP KAEKKVPASK DGGGDKKGKK KSKKSVETYK IYIFKVLKQV

61 HPDIGISSKA MSIMNSFIND IFEKLAGESA KLARYNKKPT ITSREIQTSV RLVLPGELAK

121 HAVSEGTKAV TKFTSA

**BAK01750.1**

1 MAPKAEKKPA AK**KPVEEEPA** **AEKAEK**TTAG KKPKAEKRLP AGKSAAKEGG KAKKKAKKSV

61 ETYKIYIFKV LKQVHPDIGI SSKAMSIMNS FINDIFEKLA GEAAKLARYN KKPTITSREI

121 QTSVRLVLPG ELAKHAVSEG TKAVTKFTSS

**BAK01633.1**

1 MAPKAAEKKP VEKKPKAEKK VPASK**EGGGG** **EK**KGKKKAKK SVETYKIYIF KVLKQVHPDI

61 GISSKAMSIM NSFINDIFEK LAGESAKLAR YNKKPTITSR EIQTSVRLVL PGELAKHAVS

121 EGTKAVTKFT SA

**BAK06478.1**

1 MAPKAEKKPA AK**KPAEEEPT** **TEKAEKAPAA** **K**KPKAEKRLP AGK**TASKEGG** **EK**KGRKKGKK

61 SVETYKIYIF KVLKQVHPDI GISSKAMSIM NSFINDIFEK LAGEAAKLAR YNKKPTITSR

121 EIQTSVRLVL PGELAKHAVS EGTKAVTKFT SS

**BAJ98393.1**

1 MAPKAAEKKP VEKTPAGKKP KAEKKVPASK EGGDKKGKKK SKKSVETYKI YIFKVLKQVH

61 PDIGISSKAM SIMNSFINDI FELAKHAVSE GTKAVTKFTS S

**H3**

**BAJ92605.1**

1 MARTKQTARK STGGKAPRKQ LATKAAR**KSA** **PTTGGVK**KPH R**YRPGTVALR** EIRKYQKSTE

61 LLIRKLPFQR LVREIAQDFK TDLRFQSHAV LALQEAEAYL VGLFEDTNLC AIHAKRVTIM

121 PKDIQLARRI RGERA

**BAJ96321.1**

1 MACTKQTARK STDGKAPRKQ LATKAARKSA PATDGVKKLH RFRSGTVALR EIRKYQKSTE

61 LLIRKLPFQR LVREIAQDFK TDLRFQSSAV SALQEATEAY LVGMFDDTNI CAIHVKRVTI

121 MPKDIQLVCR IRGERA

**AAB03541.1**

1 MARTKQTARK STGGKAPHKQ LATTAARKSA RTTGRLKKLH R**YRPGTVALR** EIRKYQKSTE

61 LLIRKLPFQR LVREIAQDFK TDLRFQSHAV LALQEAAEAY LRGLFEDTNL CAIHAKRVTI

121 MPKDIQL

**BAJ85229.1**

1 MARTKQTARK STGGKAPRKQ LATKAAR**KSA** **PTTGGVK**KPH R**YRPGTVALR** EIRKYQKSTE

61 LLIRKLPFQR LVREIAQDFK TDLTRSIHQP YIINHHHHQQ QLG

**AEK21392.1**

1 MARTKHPAVR KSKAPPKKKI GSASSPSAAQ R**RQETDGAGT** **SETPR**RAGRG PAPAAAEGAP

61 GEPTKRKPHR FRPGTVALRE IRKYQKSVNF LIPFAPFVRL VREITEYYCP RVKRWTPQAL

121 LAVQEATEYH LVDIFERAHL CAIHAKRVTV MQKDMQLA

**AEK21393.1**

1 MARTKKTVAA KEKRPPCSKS EPQSQPKKKE KRAYRFRPGT VALREIRKYR KSTNMLIPFA

61 PFVRLVRDIA DNLTPLSNKK ESKPTPWTPL ALLSLQESAE YHLVDLFGKA NLCAIHSHRV

121 TIMLKDMQLA RRIGTRSLW

**P06353.1**

1 KSTELLIRKL PFQRLVREIA QDFKTDLRFQ SHAVLALQEA AEAYLVGLFE DTNLCAIHAK

61 RVTIMPKDIQ LARRIRGERA

**H4**

**BAK03333.1**

1 MAACTSQHAL VSVKSRPASA SFSRDKRAGN ARFVSAAGCC PSSRKLGLVC ASNSQSSVIE

61 PAQLPSSPES GSTPKKSSEA ALILIRHGES LWNEKNLFTG CVDVPLTPKG VNETIEAGKR

121 ICNIPVDVIY TSSLIHAQMT AMLAMMQHRR KKVPIIVHKE SERAHRWSQV YSEETKKQSI

181 PVITAWQLNE RMYGELQGLN KQETADLFGK DQVHEWRRSY DIPPPNGESL EMCAERAVSY

241 FKDQIIPQLV AGKHVMIAAH GNSLRSIIMH LDKLTSQEVI SLELSTGIPM LYIFKEGKFI

301 RRGSPAGPSE AGVYDICDGR FGPRGPNRPS RPATAATHLQ RHIHRREEER SEKMSGRGKG

361 GKGLGKGGAK RHRK**VLRDNI** **QGITKPAIRR** LARRGGVK**RI** **SGLIYEETRG** **VLKIFLENVI**

421 **RDAVTYTEHA** **R**R**KTVTAMDV** **VYALKR**QGR**T** **LYGFGG**

**BAK00005.1**

1 MAGQGKGGKG VGGKGKVGTK RTAQKRHARA SIEGITKPAI RRLARRGGVK RISSFIYDDS

61 RQVLKGFLEG IVR**DAVTYTE** **HARRKTVTAM** **DVVYALKR**QG R**TIYGFGG**
