# Supplementary figures and images for: The Hypoxic Proteome and Metabolome of Barley (Hordeum vulgare L.) with and without Phytoglobin Priming
Source: Int J Mol Sci. 2020 Feb 24;21(4):1546. doi: 10.3390/ijms21041546 (PMC7073221; doi:10.3390/ijms21041546)

A

WT WT24 HO HO24

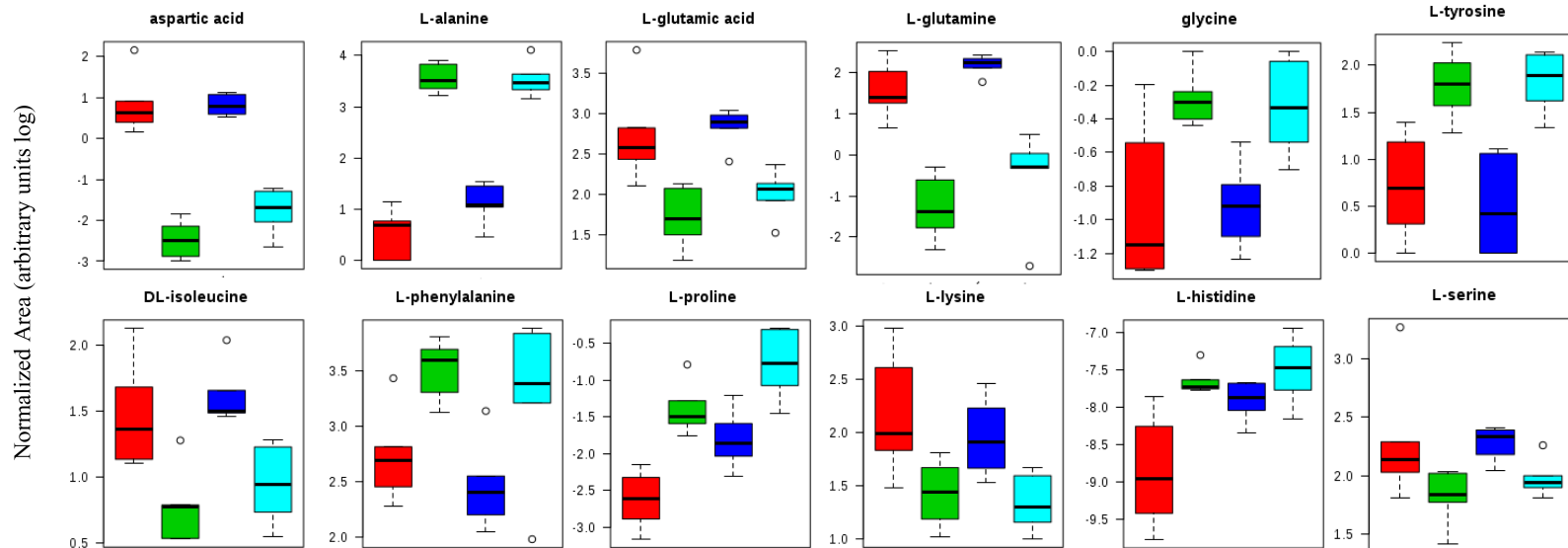

B

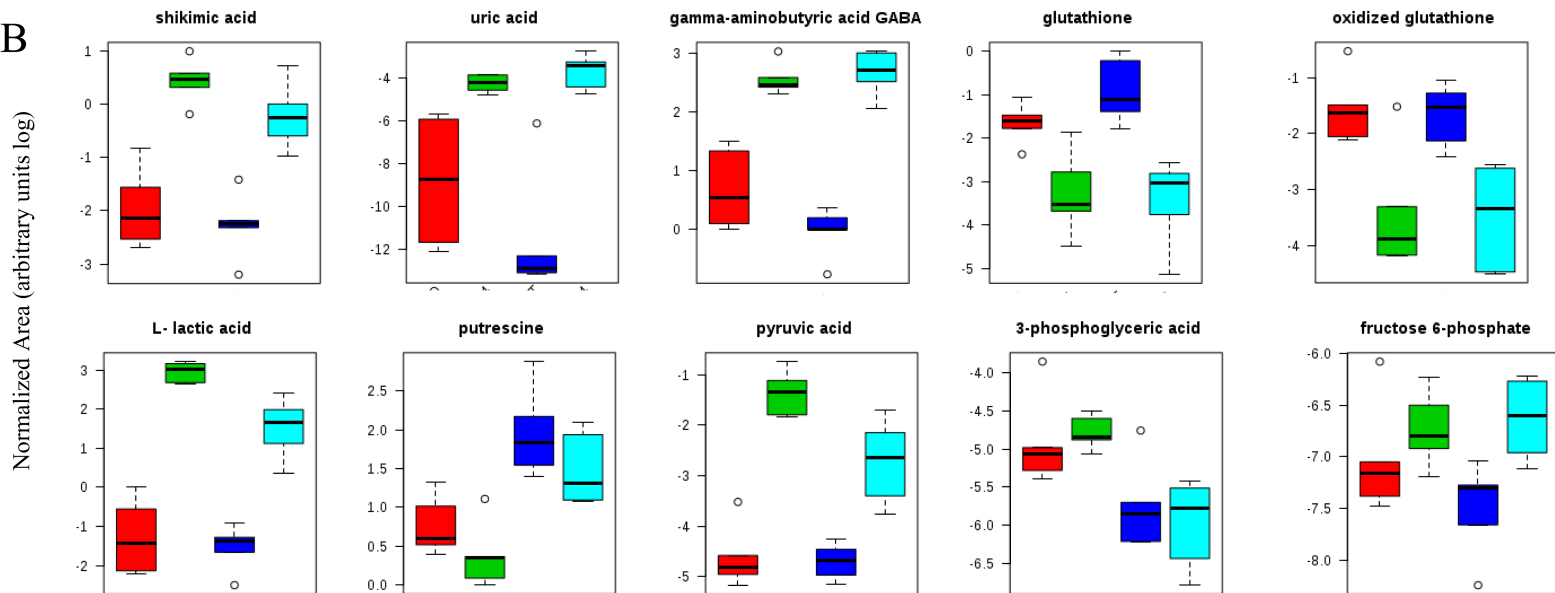

Supplement: Supplementary file 1 [file ijms-21-01546-s001.zip › ijms-726488-SI-to conversion/Figure S1_amino.pdf]
